# Stimuli-Responsive Gene Delivery Nanocarriers for Cancer Therapy

**DOI:** 10.1007/s40820-023-01018-4

**Published:** 2023-02-08

**Authors:** Qingfei Zhang, Gaizhen Kuang, Wenzhao Li, Jinglin Wang, Haozhen Ren, Yuanjin Zhao

**Affiliations:** 1 Department of Hepatobiliary Surgery, The Affiliated Drum Tower Hospital of Nanjing University Medical School, Hepatobiliary Institute of Nanjing University, Nanjing, 210008 People’s Republic of China; 2 Oujiang Laboratory (Zhejiang Lab for Regenerative Medicine, Vision and Brain Health), Wenzhou Institute, University of Chinese Academy of Sciences, Wenzhou, 325001 People’s Republic of China; 3 State Key Laboratory of Bioelectronics, School of Biological Science and Medical Engineering, Southeast University, Nanjing, 210096 People’s Republic of China; 4 Chemistry and Biomedicine Innovation Center, Nanjing University, Nanjing, 210023 People’s Republic of China

**Keywords:** Stimuli-responsive, Nanocarrier, Gene therapy, Gene delivery, Cancer

## Abstract

Gene therapy provides a promising approach in treating cancers with high efficacy and selectivity and few adverse effects. Currently, the development of functional vectors with safety and effectiveness is the intense focus for improving the delivery of nucleic acid drugs for gene therapy. For this purpose, stimuli-responsive nanocarriers displayed strong potential in improving the overall efficiencies of gene therapy and reducing adverse effects via effective protection, prolonged blood circulation, specific tumor accumulation, and controlled release profile of nucleic acid drugs. Besides, synergistic therapy could be achieved when combined with other therapeutic regimens. This review summarizes recent advances in various stimuli-responsive nanocarriers for gene delivery. Particularly, the nanocarriers responding to endogenous stimuli including pH, reactive oxygen species, glutathione, and enzyme, etc., and exogenous stimuli including light, thermo, ultrasound, magnetic field, etc., are introduced. Finally, the future challenges and prospects of stimuli-responsive gene delivery nanocarriers toward potential clinical translation are well discussed. The major objective of this review is to present the biomedical potential of stimuli-responsive gene delivery nanocarriers for cancer therapy and provide guidance for developing novel nanoplatforms that are clinically applicable.

## Highlights

- Stimuli-responsive gene delivery nanocarriers (GDNCs) possess huge potential in the gene therapy field owing to their effective protection, prolonged blood circulation, selective and targeted delivery, and controlled release of nucleic acid drugs.
- Recent advances in stimuli-responsive GDNCs for cancer therapy are classified, summarized, and exhibited by representative examples.
- The potential challenges and prospects of stimuli-responsive GDNCs toward clinical translation are outlined and the future research directions are outlooked.

## Graphical abstract

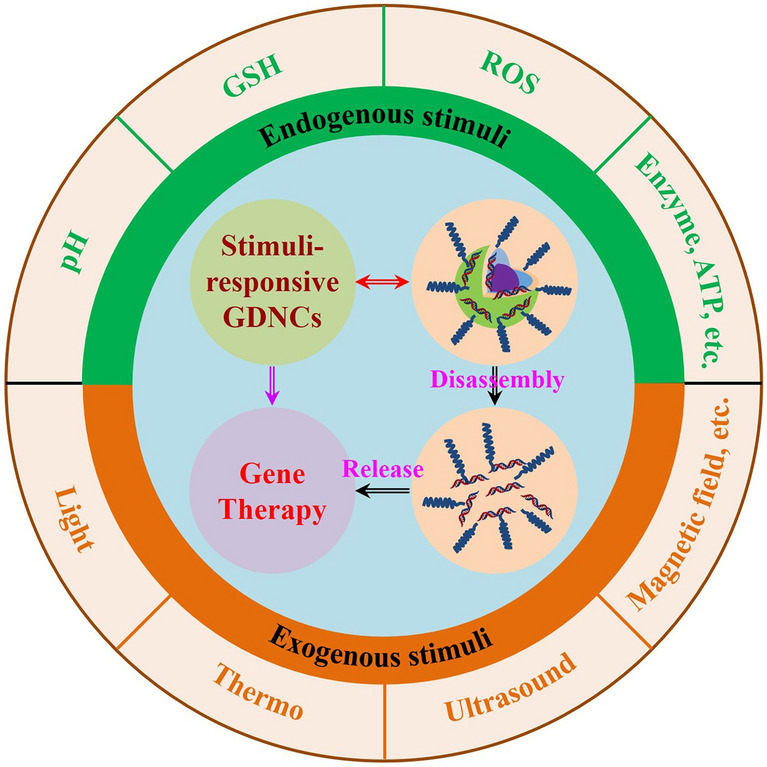

## Introduction

Cancer is one of the deadliest diseases in the world, which seriously threatens human health and life [1–7]. Tumorigenesis and progression involve the abnormal expression and mutation of a variety of genes, which are often participated in cell proliferation, differentiation, aging, death, and other processes [8, 9]. Currently, gene therapy as an emerging revolutionary treatment technology can specifically regulate and amend the expression of any aberrant gene in cancer cells in theory [10, 11]. Compared with other therapeutic modes, gene therapy has high efficiency, high selectivity, low side effects, as well as no drug resistance [12, 13]. Meanwhile, the strategy of combing gene therapy with other therapy methods can simultaneously act on different targets in cancer cells to achieve synergistically enhanced therapeutic efficacy [14]. Therefore, gene therapy has attracted numerous attention of researchers to clinicians for cancer therapy [15]. During the past decades, more than 4600 cases of gene therapy clinical trials have been conducted worldwide, 29% of which have entered the phase II/III/IV clinical trial stage for the treatment of cancers [16, 17].

The major nucleic acid drugs for the treatment of cancers include small interfering RNA (siRNA) [18–20], antisense oligonucleotides [21, 22], microRNA [23, 24], messenger RNA [25–29], short hairpin RNA, and gene editing systems (e.g., CRISPR/Cas9 system) [30–32]. Each kind of these nucleic acid drugs can specifically work on target cells at the genetic level in its own way. However, the naked nucleic acid drugs cannot achieve the desired outcomes when administrated directly due to their non-specific biological distribution, low cellular uptake, rapid clearance, and enzymatic degradation [33, 34]. To solve this dilemma, physical methods (nuclear transfection, electroporation, etc.) and chemical methods (viral vector and non-viral vector) have been elaborately explored for nucleic acid drug delivery [35]. Unfortunately, nuclear transfection and electroporation can cause great damage to cells, which limits their application in vivo [36]. For another, although the pathogenicity is eliminated and the gene transfection ability is retained, the commonly used viral vectors such as adenovirus, adeno-associated viral, and retrovirus face some problems such as immunogenicity and biosafety, difficult preparation, limited packaging capacity, and poor target specificity [37]. By contrast, because of their many advantages including easy preparation, low toxicity, low immunogenicity, high gene packaging capacity, and unrestricted gene loading type, non-viral vectors (such as cationic polymers, cationic liposomes, inorganic or organic nanoparticles) have been brought into research focus on gene delivery [38–40]. Particularly, nanocarrier-based vectors have been widely developed to achieve specific clinical goals that cannot be accomplished by traditional gene delivery methods [41, 42]. Owing to their many merits including i) improving stability and preventing premature degradation of genes, ii) improving pharmacokinetics, prolonging drug circulation time, and controlling gene release behavior, iii) achieving selectively and targeted gene delivery to the lesion site, iv) incorporating multiple genes and imaging agents for combined therapy and tracking, the gene delivery nanocarriers (GDNCs) have been rapidly developed in recent years [43–50].

**Fig. 1:**
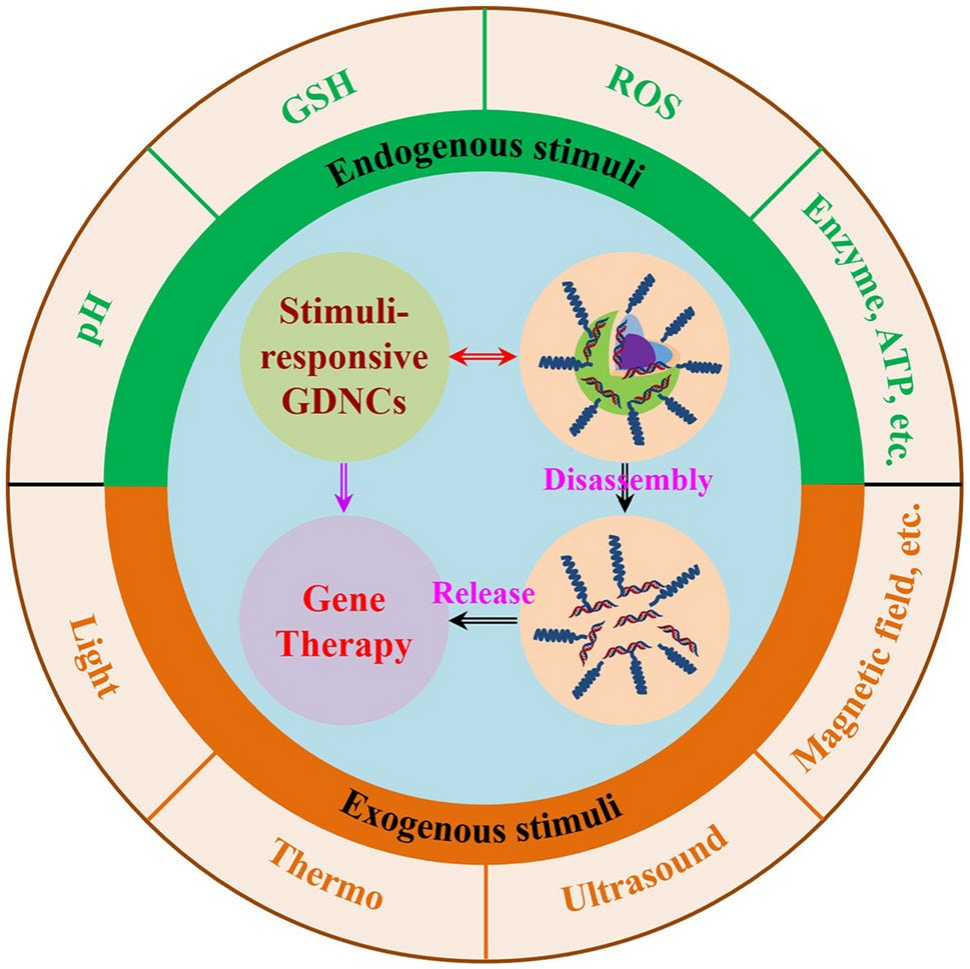
Schematic diagram of stimuli-responsive nanocarriers for gene delivery and cancer therapy

Despite great progress, in the face of the complicated intrinsic tumor environment, mediocre nanocarriers often seem beyond their grasp for achieving promising therapeutic potential [51]. Thus, intelligent GDNCs are highly sought-after. Recently, stimuli-responsive GDNCs that could efficiently deliver nucleic acid drugs to targeted sites and respond to a specific stimulus for maximizing the efficiency of gene therapy, have attracted much attention [52–55]. These GDNCs mainly include those nano-vectors that can be activated by endogenous stimuli (pH, reactive oxygen species (ROS), glutathione (GSH), enzyme, etc.) [56–60] and exogenous stimuli (light, thermo, ultrasound (US), magnetic field (MF), etc.) (Fig. 1) [61–65]. Considering their unique merits, this review introduces an outline of the successes and challenges of the stimuli-responsive GDNCs for gene delivery in cancer therapy. The advancements of various types of stimuli-responsive GDNCs are classified and summarized, and the perspectives and prospects in this area are discussed.

## Various Stimuli

The stimuli that are commonly employed to elaborately design novel stimuli-responsive GDNCs mainly come from intrinsic endogenous tumor microenvironment (TME) and artificial exogenous conditions. The TME typically exhibits great differences in physical and chemical characteristics compared with normal tissues, such as reduced pH, high-level ROS and GSH, overexpressed enzymes, hypoxia, and elevated triphosadenine (ATP) [66–68]. In addition to promoting tumor angiogenesis and metastasis, the TME could induce treatment resistance and failure. Therefore, to manage the release of nucleic acid drugs used in gene therapy, the TME is frequently utilized as a hallmark for cancer treatment, while light, thermo, US, MF, etc., [69–72] are the mainly artificial exogenous stimuli that are applied for developing various GDNCs in gene therapy. By utilizing these exogenous stimuli, timely, localized, and quantitative gene release might be achieved.

### pH

Numerous studies have demonstrated that the Warburg effect, a disordered energy metabolism, will cause poor perfusion and the accumulation of lactic acid, resulting in weakly acidic (pH range from 6.5 to 6.8) extracellular space of tumor tissue [73, 74]. The pH value in the internal lysosomal and endosomal compartments, which ranges from 4.5 to 6.5, is much lower than that in the TME [75, 76]. Therefore, compared with the neutral environment of normal tissues, the high extracellular acidity of tumors has been widely utilized as a common pathological characteristic for the construction of acid-activated nanodrugs or nanoprobes [77–79]. For instance, cis-maleic monoamides could be destroyed entirely at pH 6.5 after several hours and change surface charge, while they were moderately stable at pH 7.4 [80]. Due to the propensity to protonate in acidic environments, tertiary amine groups containing nanomedicine demonstrate a typical pH-responsive property. Additionally, several acid-labile linkers have been thoroughly investigated for the creation of pH-responsive nanomaterials, including maleimide, cis-aconityl, and hydrazones [81]. The acid-sensitive chemical linkers will be broken when the pH-responsive nanocarriers reach the tumor site and undergo changes in pH. Representatively, the nanocarrier's electrical characteristics, size, and hydrophilicity are triggered to transform [82]. Thus, the exploration and application of pH-responsive GDNCs are highly anticipated.

### GSH

GSH is a tripeptide with a thiol group and a γ-amide bond and is the most widespread thiol in mammalian cells [83, 84]. GSH is crucial for maintaining the cell membrane's integrity, the redox state's equilibrium, and the thiol-containing enzymes' stability [51]. Generally, the concentration of GSH in the extracellular matrix (ECM) or blood is between ~ 2 and 20 µM. However, the concentration of GSH is dramatically up-regulated to approximately 1000 times higher than that in the blood or ECM because of the fast multiplication of tumor cells [85]. Therefore, GSH can be utilized as a specific marker to enable the development of GSH-responsive drug delivery systems (DDSs). GSH could quickly cleave the disulfide bond (S–S) and the diselenide bond (Se-Se), making them typical building blocks for reduction-responsive carriers [86, 87]. Generally, in the extracellular environment, S–S and Se-Se are stable with low GSH concentrations [88]. They can be cut off in the intensely reducing intracellular space by the GSH-mediated thiol-disulfide or selenol-diselenide exchange process, leading to the cleavage of the carrier and release of the cargo [89]. Recently, platinum(IV) (Pt(IV)) prodrugs are also found to be sensitive to reductive molecules in tumor cells, and the Pt(IV)-based nano-DDSs have been widely investigated for effective cancer treatment [90–92]. Thus, the reduction-responsive GDNCs are also popular in developing highly effective gene therapy systems.

### ROS

In general, ROS in organisms refers to oxygen-derived free radicals and non-free radicals, including superoxide anion (O_2_^•^), hydrogen peroxide (H_2_O_2_), hydroxyl radical (OH^•^), and singlet oxygen (^1^O_2_), which is essential to diverse physiological processes [93–95]. ROS levels are 100 times higher in tumor cells than in normal cells as a result of oxidative stress, which is characterized by an imbalance between ROS generation and antioxidant defense [96, 97]. Therefore, the high level of ROS in TME is a common feature that can be used to design oxidation-responsive nanoplatforms [98]. In such carriers, reductive groups are frequently present and can interact with oxidizing substances like chalcogen (sulfur bonds, selenium bonds, tellurium bonds, aromatic borate bonds, peroxalate bonds, etc.) [99]. Thioethers are another often utilized reductive group that could be reduced to ketones. Selenium, a vital element of GSH peroxidase, can shield cells from oxidative stress [100]. Selenium bonds deteriorate more quickly than sulfur compounds, and the diselenide bonds can undergo bidirectional reactions with both reducing and oxidizing agents [101]. By utilizing the ROS-sensitive chemical bonds or molecules, stimuli-responsive GDNCs could be obtained.

### Light

Light is a frequently used exogenous stimulus in the clinical application of various diseases [102–105]. Due to the advantages of spatiotemporal selectivity, remote controllability, noninvasiveness, and convenient operation, light-responsive DDSs based on phototherapies including photoactivated chemotherapy [106, 107], photodynamic therapy (PDT) [108–111], photothermal therapy (PTT) [112–114], etc., have been broadly used for cancer treatment [115, 116]. By using the light-controllable DDSs, on-demand drug/gene/protein release could be achieved for precision strikes against cancer cells. In the past decades, many light-activated molecules such as azobenzene derivatives, spiropyran derivatives, and *o*-nitrobenzyl moieties have been employed in the light-responsive DDSs [117–119]. Some photo-activatable prodrugs like platinum(IV)-azide complexes have also attracted great attention [120–124]. As relevant studies conducted, the light-responsive GDNCs based on light-activated chemical bonds, molecules, or prodrugs could be applied for improving the therapeutic effect of gene therapy alone or combined with other treatment modalities.

### Other Stimuli

Endogenous stimuli such as the enzyme and ATP are also commonly used in the design of stimuli-responsive nanocarriers [125, 126]. Enzymes are proteins or RNAs generated by living organisms. They have a high level of specificity and catalytic activity for their substrate [127]. It has been found that the secretion of enzymes in TME is excessive in comparison with that of normal tissues [128, 129]. For example, matrix metalloproteinases-2 (MMP-2) expression levels in MDA-MB-231 cells were found to be almost 6 times higher compared to normal HS578Bst cells [130]. Nearly 8 times more hyaluronidase (HAse) was expressed in high-grade bladder cancer than in regular bladder tissue [131–133]. ATP is a kind of high-energy phosphate compound. The inter-transform of ATP and adenosine diphosphate can realize energy storage and exergy, to ensure the energy supply of various life activities of cells [134]. Studies have shown that ATP concentrations vary between normal and malignant cells, which has inspired the development of ATP-responsive nanomaterials [135].

Thermo, US, and MF are other exogenous stimuli [136–140]. Their spatiotemporal controllability and noninvasive manipulation of drug/gene/protein delivery promote the development of the corresponding stimuli-responsive DDSs. Specifically, based on the thermo-sensitive lipid molecules and chemical bonds, thermo-responsive GDNCs would be well designed [141–143]. The US can penetrate deep tissues with high precision and can control the release of bioactive molecules without causing harm to nearby healthy tissues [144]. Besides, US-activated ROS from sonosensitizers can be used to trigger the disassembly of ROS-sensitive nanocarriers and for sonodynamic therapy (SDT) [145]. Unlike chemical and optical signals, the MF is not attenuated by tissue and has no obvious negative effects on the human body [146, 147]. The development of magnetic-responsive GDNCs also enables spatial control gene therapy. All in all, the mentioned various stimuli inspire the development and application of stimuli-responsive GDNCs, which will bring great hope to the field of gene therapy against tumors and other diseases.

## Stimuli-Responsive GDNCs

### pH-Responsive GDNCs

Acidic TME inspires the broad development of pH-responsive nano-DDSs for cancer therapy [51, 148]. For this aspect, the pH-responsive GDNCs based on the concept of charge reversal strategy is one of the commonly used systems in nucleic acid drug delivery [149]. Most proteins in plasma are negatively charged, imparting them the capability of adsorbing nanoparticles with positive surface charges [150]. As a result, the positively charged nanoparticles are readily detected and eliminated from the blood by phagocytic cells, which significantly lowers the bioavailability of drugs. By engineering the surface to be negative or neutral, the circulation time of GDNCs in the body could be extended. However, the electronegative nature of the cell membrane makes it difficult for cells to absorb GDNCs with negative surface charges. On the contrary, the positively charged GDNCs are easily endocytosed by cells. Therefore, intelligently regulating the surface charge of GDNCs at different stages through rational design is important.

**Fig. 2:**
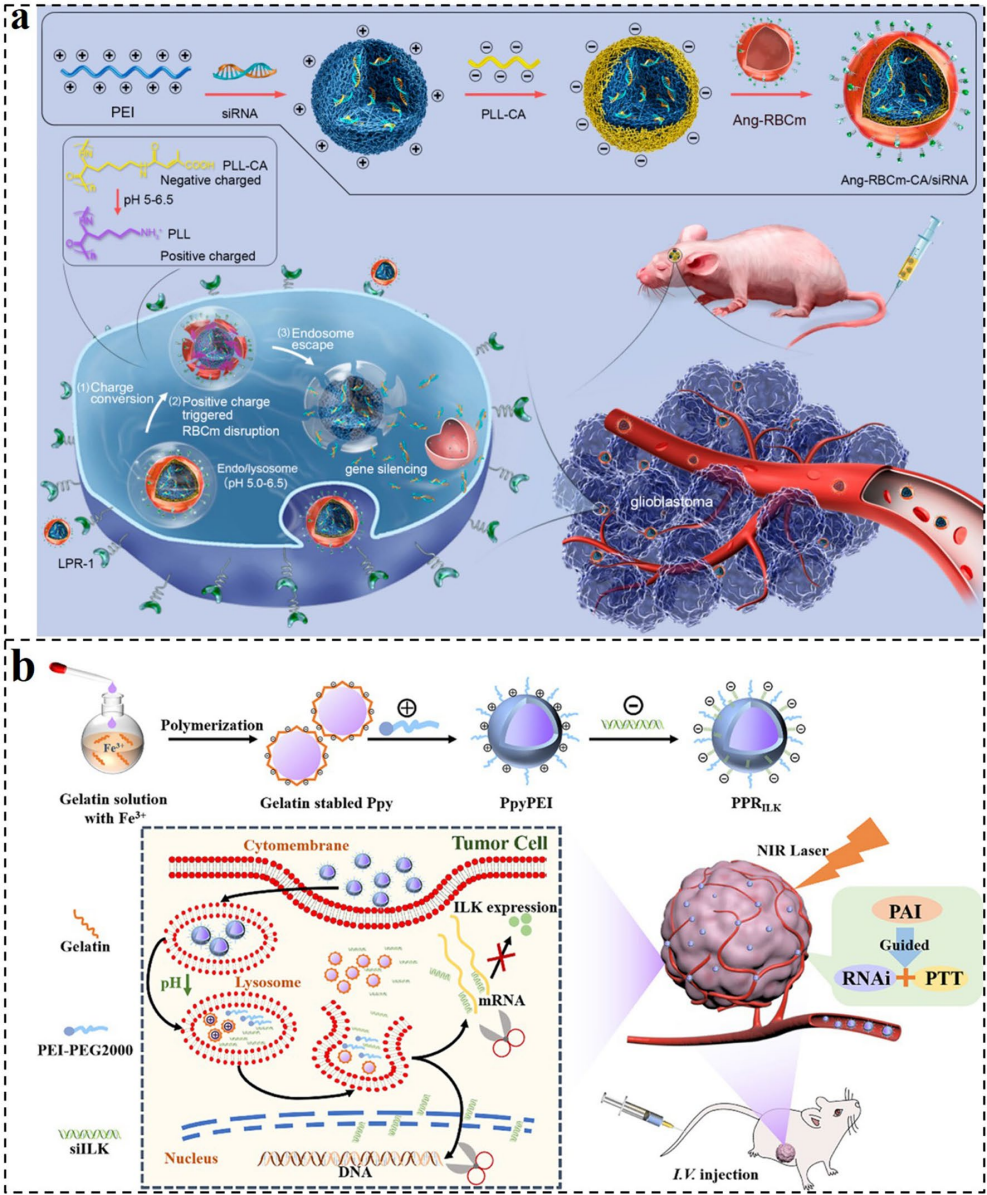
**a** Preparation and therapeutic processes of Ang-RBCm-CA/siRNA. Ang-RBCm-CA/siRNA boasts a long circulation time, enhanced BBB penetration, efficient cell uptake via LPR overexpressing U87MG glioblastoma cancer cells, charge conversion, and membrane disruption triggered by endosomal low pH and siRNA release, culminating in the effective treatment of orthotopic U87MG human glioblastoma in tumor-bearing nude mice [151]. Copyright 2020, American Chemical Society. **b** Illustration of the fabrication and structure of the PPR_ILK_ nanocomplex and its functions for combined photothermal and gene therapy in PTC [152]. Copyright 2022, American Chemical Society

As one paradigm, Shi et al. designed a charge reversal siRNA nanocomplex (Ang-RBCm-CA/siRNA) for efficient siRNA delivery and glioblastoma (GBM) RNA interference (RNAi) therapy (Fig. 2a) [151]. The Ang-RBCm-CA/siRNA consisted of polyethylenimine (PEI) and siRNA complexes as the inner core, poly-L-lysine grafted with citraconic anhydride (PLL-CA) as the middle layer, and red blood cell membrane modified with Angiopep-2 (Ang-RBCm) as out-layer. The unique three-layer core–shell structure nanocomplex demonstrated increased blood–brain barrier (BBB) transcytosis, extended blood circulation time, and efficient tumor-selective targeting. Particularly, after the endocytosis of Ang-RBCm-CA/siRNA, the PLL-CA layer can be triggered to cleave and expose a positive charge in the endosome, resulting in the RBCm rupture and efficient siRNA unpacking. Of note, the nanocomplex of Ang-RBCm-CA/siRNA exhibited considerable effective transfection ability to the commercial Lipofectamine 2000 with non-toxic and good biocompatibility. The Ang-RBCm-CA/siPLK1 demonstrated a significant tumor inhibition of 82.4%, according to the in vivo antitumor results. Thus, this multi-functional nanoplatform could significantly improve the siRNA delivery and showed excellent therapeutic efficiency on GBM, providing an effective and functional system for gene therapy.

Based on a similar strategy, Wang et al. reported charge reversal GDNCs for gene delivery and synergistic PTT (Fig. 2b) [152]. The nanocarrier (PPR_ILK_) combining PTT, photoacoustic imaging (PA), and RNAi functions was fabricated by gelatin-stabled polypyrrole (Ppy) − PEI − siILK via electrostatic self-assembly technology. The shell of gelatin-stabled Ppy displayed a negative charge under the normal physiological environment with pH 7.35 − 7.45, and turned into a positive charge when the pH decreased to 4.0 − 5.5. Therefore, the PEI and siILK adsorbed by gelatin-stabled Ppy would be released from the PPR_ILK_ nanocarrier after being endocytosed by tumor cells and enwrapped into lysosomes with an acidic environment. Then, the siILK could further escape from lysosomes via the “proton sponge effect (PSE)” to the cytoplasm, thus performing its function in inhibiting the proliferation of papillary thyroid cancer (PTC) and lymphatic metastasis. Together with Ppy-mediated PTT, fluorescence, and PA bimodal imaging, the PPR_ILK_ nanocarrier could efficiently eradicate PTC and prevented recurrences and metastases.

**Fig. 3:**
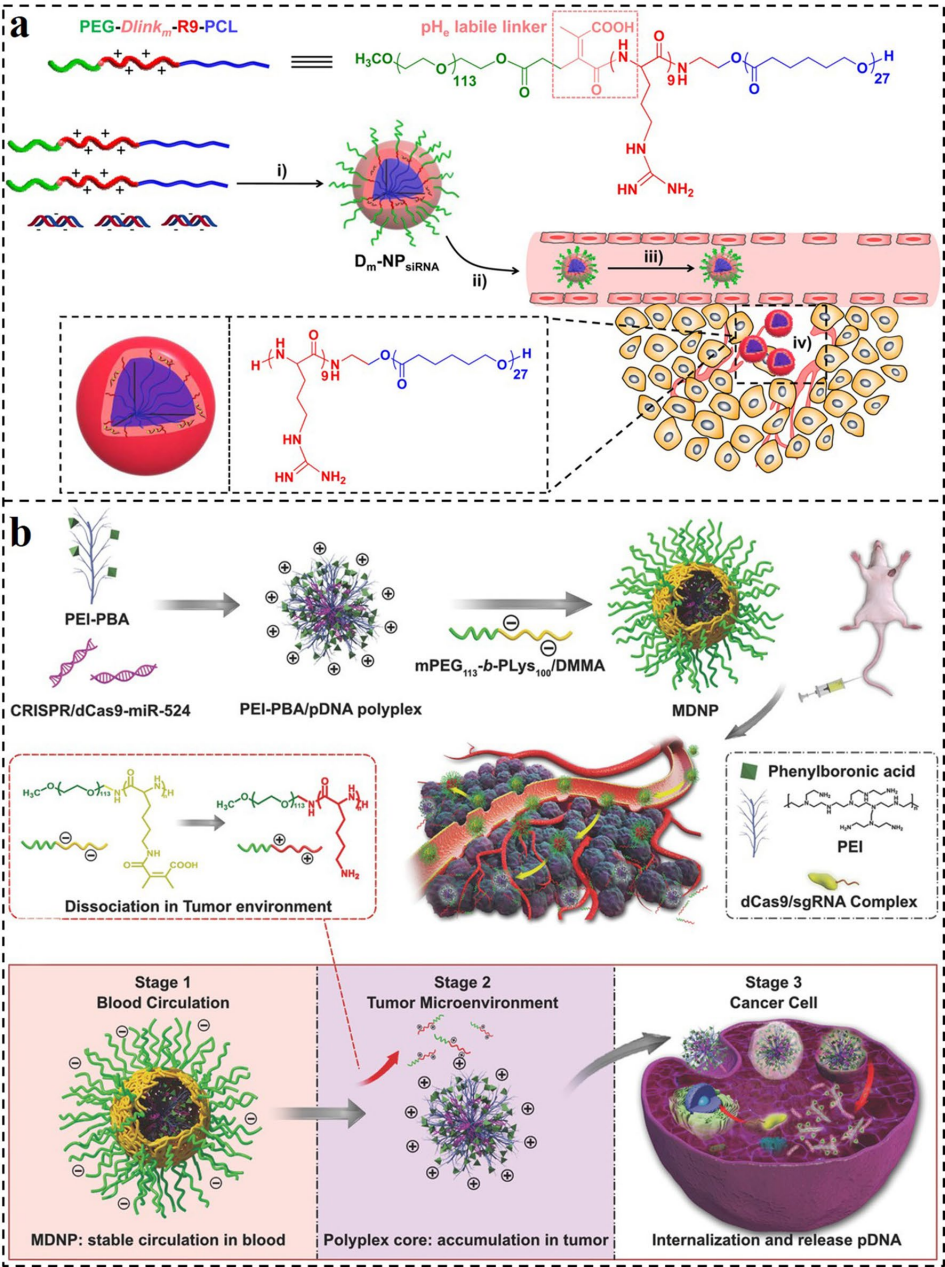
**a** Illustration of the self-assembly of PEG-*Dlink*_*m*_-R9-PCL, formation of D_m_-NPsiRNA, and systemic administration of D_m_-NPsiRNA [153]. Copyright 2015, American Chemical Society. **b** Schematic illustration for the preparation of MDNP and delivery process after intravenous injection [154]. Copyright 2018, Wiley–VCH GmbH

In addition, Wang and colleagues reported an acid-sensitive micelleplex delivery system (D_m_-NP) for active targeted siRNA delivery (Fig. 3a) [153]. The D_m_-NP self-assembled from PEG-*Dlink*_*m*_-R9-PCL could bind with siRNA to form D_m_-NP_siRNA_. This delivery system could prolong the circulation of siRNA and protect siRNA from reticuloendothelial system (RES) clearance with the protection of an extremely stable poly(ethylene glycol) (PEG) out-layer. After accumulation into tumor sites, the degradable bridged bond linkage (*Dlink*_*m*_) could respond to the low pH in the tumor (pH_e_) and cleaved to detach the PEG corona for cell targeting. Meanwhile, the exposed cell-penetration peptide of cationic nona-arginine (R9) after the separation of PEG could further enhance the cellular uptake of the complexes. Thus, the extensive and practical pH-responsive D_m_-NP_siRNA_ demonstrated higher gene silencing effectiveness and tumor suppression ability with fewer side effects, providing a promising strategy for gene delivery against cancer treatment.

In another demo, Liu and colleagues reported a charge reversal multistage nanoplatform (MDNP) for CRISPR/dCas9 system delivery and cancer therapy (Fig. 3b) [154]. The MDNP has a core–shell structure. The core of MDNP is a cationic complex composed of CRISPR/dCas9 plasmid DNA (pDNA) and phenylboronic acid (PBA)-decorated low molecular weight PEI (PEI–PBA). The shell of MDNP is a negatively charged polymer of 2,3-dimethylmaleic anhydride (DMMA)-modified poly(ethylene glycol)-b-polylysine (mPEG_113_-b-PLys_100_/DMMA). During the circulation in the blood, the negatively charged and PEGylated polymer could effectively keep the MDNP away from clearance. Then, when accumulating in tumor sites, the DMMA groups will gradually decompose under the acid TME, converting the negatively charged mPEG_113_-b-PLys_100_/DMMA to cationic one (mPEG_113_-b-PLys_100_). As a result, the shell of MDNP would gradually detach due to the electrostatic repulsion and subsequently expose the cationic polyplex core. The positively charged core with sialic acid-targeted PBA groups further enhances the accumulation and cell uptake of tumor cells. After internalization, the polyplex could escape from the endosome through PSE induced by PEI, and the pDNA would unpack from the polyplex. Finally, the loaded CRISPR/dCas9–miR-524 system significantly suppressed the growth of the tumor. With these features, this multistage delivery strategy provides a viable method for the progress and application of CRISPR-based tumor therapy technologies.

**Fig. 4:**
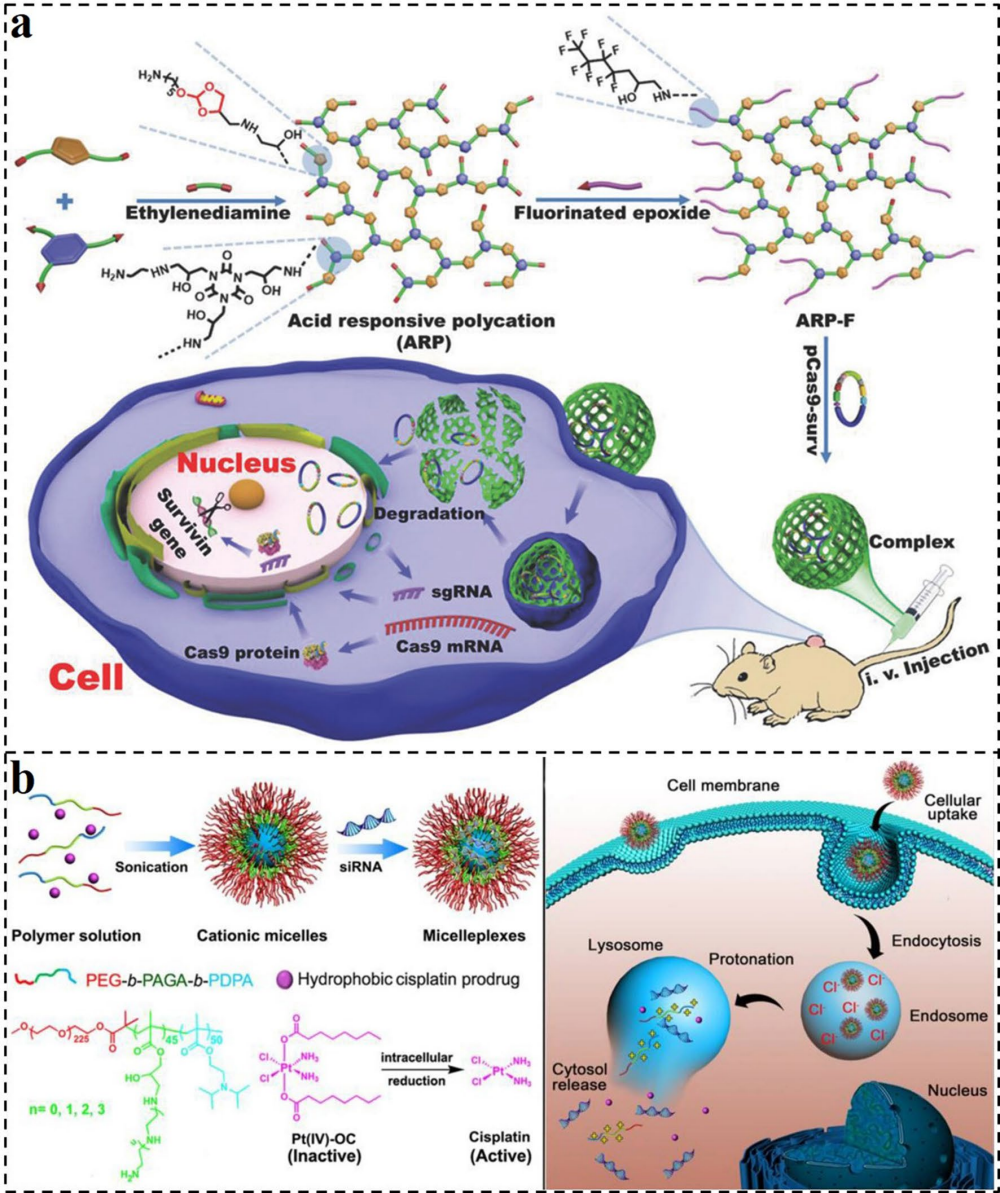
**a** Schematic illustration of the preparation of fluorinated acid-responsive polycation (ARP-F) and its resultant plasmid (e.g., pCas9-surv) delivery process [155]. Copyright 2018, Wiley–VCH GmbH. **b** Schematic illustration of the preparation and intracellular activation of siRNA and cisplatin prodrug co-loaded pH-responsive micelleplexes [156]. Copyright 2016, Ivyspring International

The combination of gene therapy and other therapeutic method is with great promise for cancer treatment because they can synergistically maximize the beneficial therapeutic effects of each therapeutic agent against cancers. Thus, the utilization of intelligent multi-functional nanocarriers for functional genes and therapeutic agent delivery has gained more attention. For example, Xu and co-workers reported an acid-labile nanosystem for CRISPR-Cas9 plasmid and temozolomide (TMZ) delivery for combination therapy of cancer (Fig. 4a) [155]. They synthesized a branched hydroxylrich polycation (ARP-F) with a positive charge and plentiful acid-sensitive ortho ester linkages. It could compact plasmids to form the acid-labile polycation/plasmid complex nanosystems. The nano-vector could effectively deliver various types of plasmids to cancer cells and then dissociate to release plasmids under the acid TME for realizing corresponding functions. Thus, the results demonstrated that the system of ARP-F/pCas9-surv could efficiently inhibit the growth of tumors. After combining TMZ and ARP-F/pCas9-surv, increased tumor therapeutic efficacy could be achieved by inducing cancer cells more sensitive to anticancer agents.

In addition, Li and co-workers reported pH-responsive nanocarriers with the triple-layered structure for siRNA and cisplatin prodrug co-delivery to enhance the therapeutic outcome of metastatic breast cancer (Fig. 4b) [156]. Firstly, the pH-sensitive triblock copolymer poly(ethylene glycol)-block-poly(aminated glycidyl methacrylate)-block-poly(2-(diisopropylamino) ethyl methacrylate) (PEG-*b*-PAGA-*b*-PDPA) was synthesized. It includes a hydrophobic PDPA core, a cationic PAGA middle layer, and a hydrophilic PEG corona. Similarly, the PEG layer could protect the siRNA-loaded nanocarriers from protein absorption and RES elimination to prolong systemic circulation. The interlayer with a positive charge could avoid the siRNA from serum degradation and promote tumor cell uptake. At the same time, the hydrophobic core could encapsulate cisplatin prodrug and stabilize the nanocomplexes during blood circulation. Particularly, the tertiary amine of the PDPA would be protonated after the nanocarriers were endocytosed into endosomes with low pH and then specifically dissociated to release siRNA and anticancer drugs. Based on these characteristics, the pH-responsive micelleplexes can prevent the migration and invasion of cancer cells and remarkably inhibit the growth and metastasis of tumors in vivo.

**Fig. 5:**
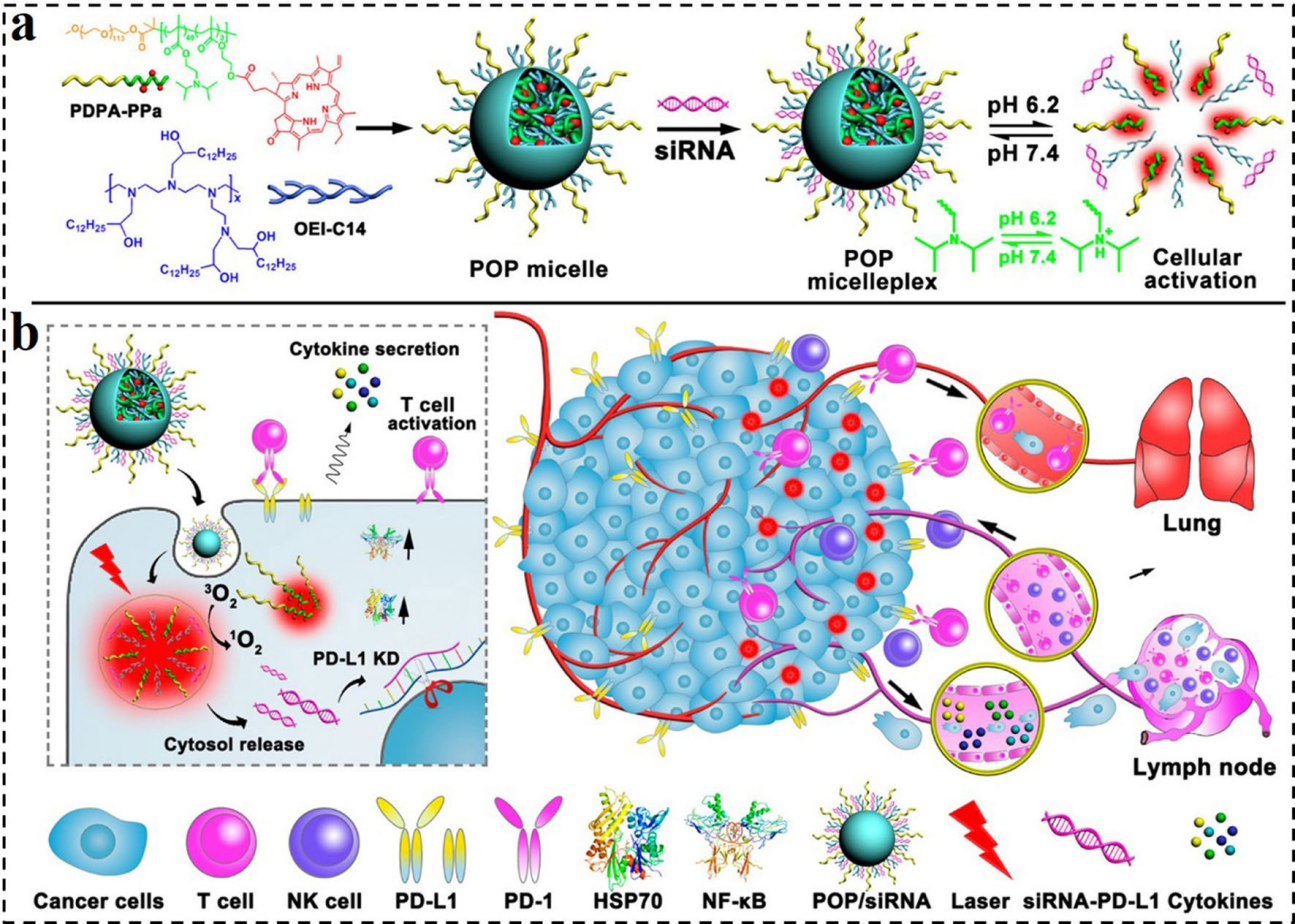
**a** Chemical structure of the acid-activatable POP micelleplexes co-loaded with PPa and siRNA. The micelleplexes can be dissociated in an acidic microenvironment due to the protonation of the tertiary amino groups of PDPA. **b** Cartoon schematic of POP − PD-L1 micelleplex-mediated photodynamic immunotherapy. The POP − PD-L1 micelleplexes induce ROS generation upon PDT. ROS consequently induces an adaptive immune response by eliciting HSP70 and NF-κB expression, triggering pro-inflammatory cytokine secretion and recruiting tumor-infiltrating T cells. The PDT-induced antitumor immune response can be further enhanced by RNAi of PD-L1 [160]. Copyright 2016, American Chemical Society

Tumor immunotherapy is a cancer therapeutic method by initiating and preserving the tumor-immune cycle and tries to restore normal systemic antitumor immune reaction [157–159]. Immune checkpoint blockade (ICB) is one of the most studied and fastest-developing immunotherapies. However, the disadvantages of low immune response rate and high treatment cost limit the therapeutic efficacy and application of ICB therapy, respectively. Downregulation of the expression of programmed cell death 1 and its ligand (PD1/PD-L1) in tumor cells by siRNA can effectively achieve ICB therapy. Herein, Li et al. rationally designed a POP polyplex composed of an ultra-pH-sensitive diblock polymer of poly(ethylene glycol)-block-poly(diisopropanol amino ethyl methacrylate-co-hydroxyethyl methacrylate)(PDPA), a photosensitizer (PS) of pheophorbide A (PPa), polycation of 1,2-epoxytetradecane alkylated oligoethylenimine (OEI-C14) and siRNA targeted to PD-L1 (Fig. 5) [160]. The POP micelleplex was inert and kept integrity at physiological conditions, which could protect the siRNA from premature leaking during circulation. However, when the micelleplexes were endocytosed by tumor cells and underwent a pH decrease in the acidic endosomes, the PS and siRNA could be triggered to release for activating fluorescence imaging, PDT, and PD-L1 interference. siRNA-mediated PD-L1 inhibition and PDT-induced immunotherapy together greatly diminished cancer progression and distant metastasis., providing a general strategy for cancer photodynamic immunotherapy.

### GSH-Responsive GDNCs

**Fig. 6:**
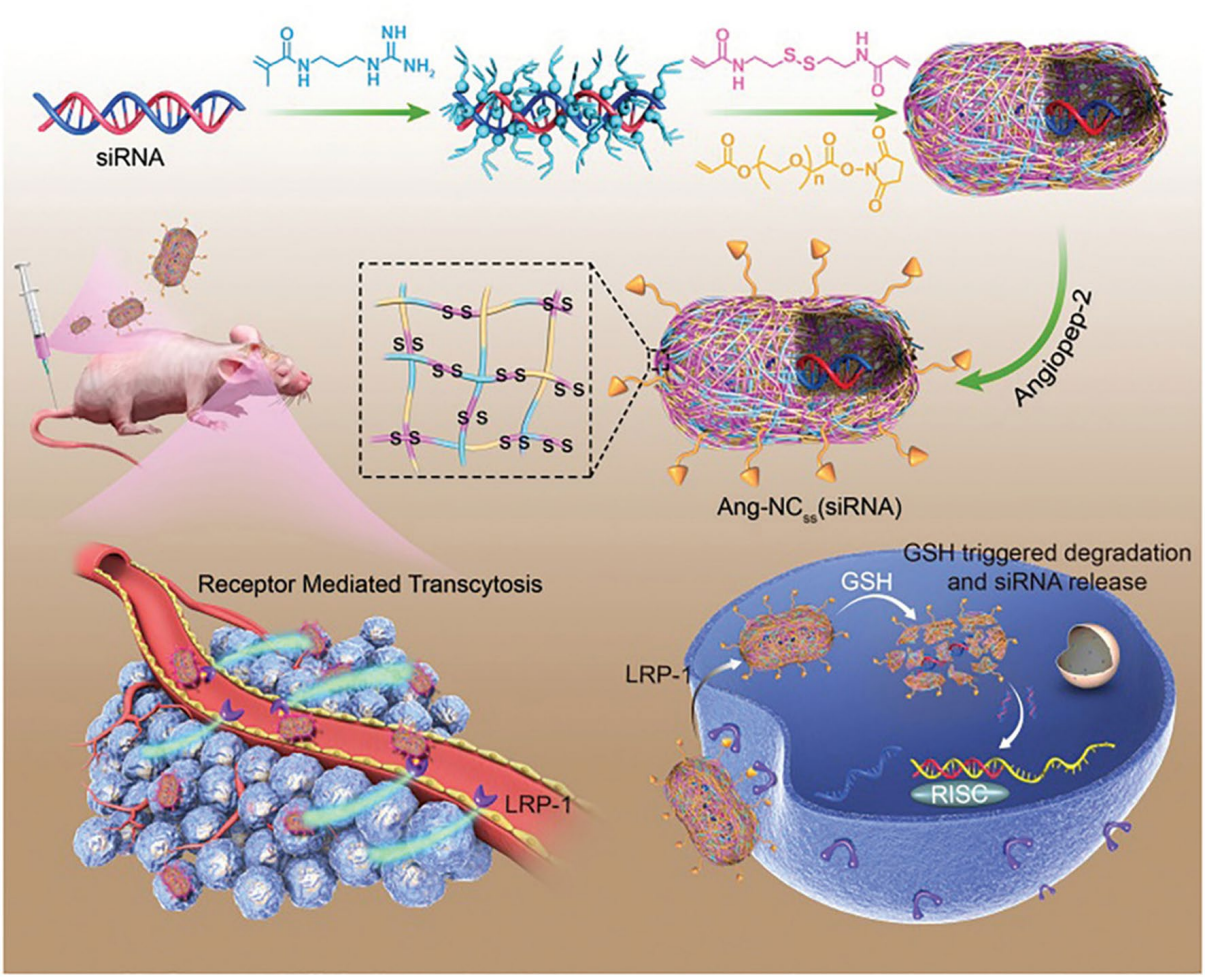
Illustration of Ang-NCss(siRNA) nanocapsule preparation, efficient BBB penetration, highly specific GBM targeting, responsive drug release, and gene silencing [161]. Copyright 2020, Wiley–VCH GmbH

Redox responsiveness has been frequently exploited as a trigger of stimuli-responsive GDNCs due to the significantly greater level of GSH in cancer cells in comparison with normal cells. S–S as one of the commonly used GSH-cleavable functional group is widely integrated into the structure of DDSs to develop GSH-sensitive nanomedicine. Based on this, Shi and colleagues designed an Angiopep-2 (Ang) decorated single nanocapsule (Ang-NC_ss_(siRNA)) for safe and efficient siRNA delivery and to achieve effective RNAi on GBM (Fig. 6) [161]. The Ang-NC_ss_(siRNA) was prepared through a self-encapsulation strategy. In brief, the acrylate guanidine was electrostatic adsorbed to siRNA before being polymerized with N, N′-bis(acryloyl) cystamine and acrylate PEG with succinate functional end groups to form NC_ss_(siRNA). Then, the targeting peptide of Ang was decorated on the nanocapsules to obtain the final Ang-NC_ss_(siRNA). The self-encapsulation strategy results in nearly 100% encapsulation of Ang-NC_ss_(siRNA) and could protect the siRNA from degradation. Subsequently, the Ang-conjugated nanocapsules could target highly expressed receptor-related protein 1 in endothelial cells and GBM tissue, promoting highly effective BBB penetration and accumulation in GBM tissue of Ang-NC_ss_(siRNA). Notably, the polymerization shell contained abundant S–S which could be reduced by a high concentration of GSH in the cytosol of cancer cells. The Ang-NC_ss_(siRNA) could induce 67% downregulation of the target gene in vitro, demonstrating effective gene knockdown capability. The in vivo studies indicated that the Ang-NC_ss_(siRNA) effectively prevented the growth of orthotopic xenografts of the U87MG tumor with good biocompatibility. Thus, the developed siRNA nanocapsules had great potential in GBM RNAi therapy and other brain-related disease therapy.

**Fig. 7:**
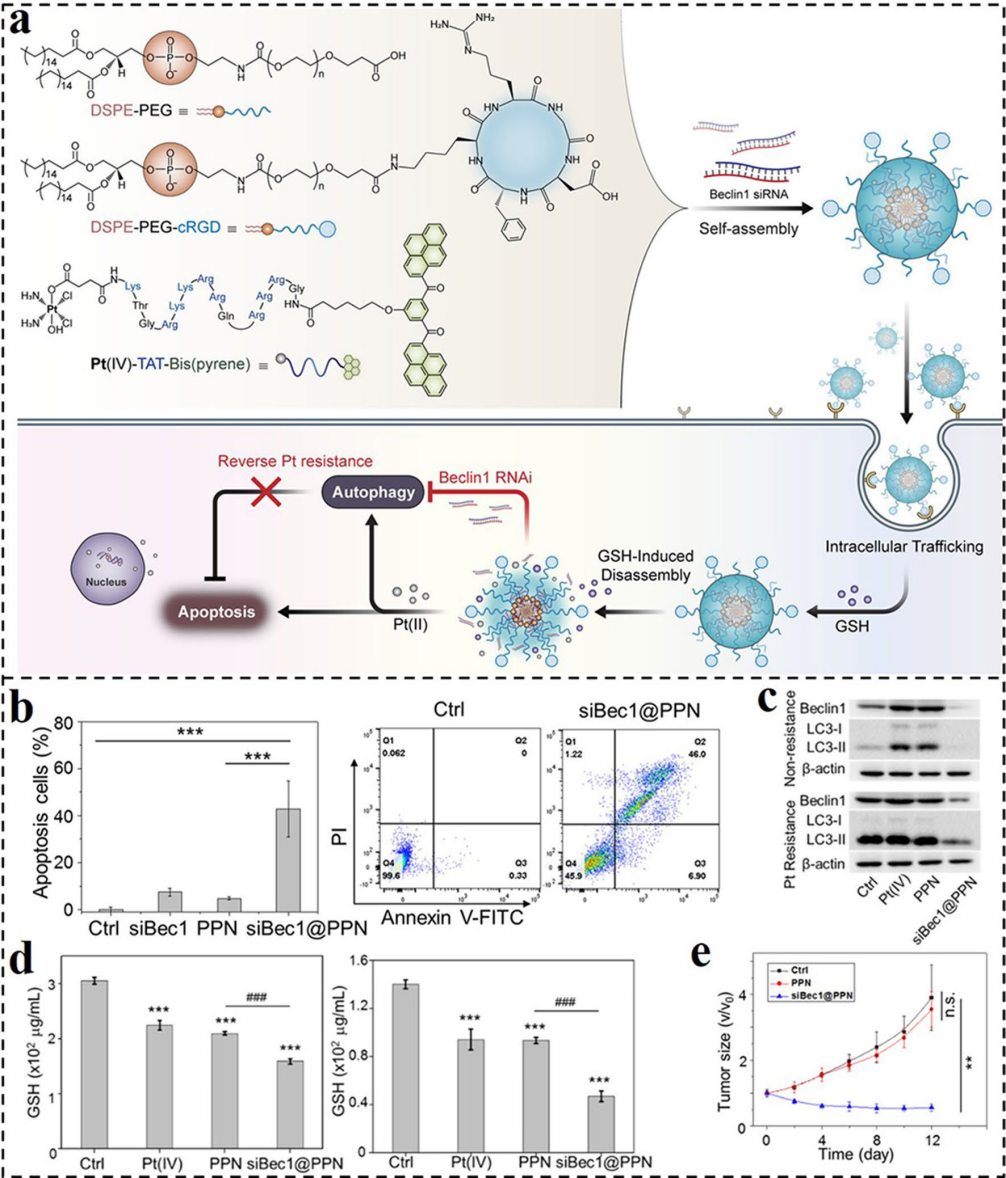
**a** Illustration of the self-assembled nano-prodrug system for co-delivery of gene and cisplatin, and the mechanism of cisplatin resistance reversion through RNAi-mediated autophagy inhibition. **b** Annexin V-FITC/PI assay for apoptosis detection in A549 tumor treated with different formulas. **c** Western blot of Beclin1 and LC3-II in A549 cells or Pt-resistant A549 cells treated with different formulas. **d** GSH level of Pt-resistant A549 cells and normal A549 cells treated with different formulas. **e** Tumor volumes of Pt-resistant A549 tumor-bearing athymic nude mice during treatment [162]. Copyright 2019, American Chemical Society

Pt(IV) prodrugs are sensitive to high concentrations of reductants in tumor cells and could be employed as a reduction-responsive group to develop stimuli-responsive DDSs for improving the therapeutic effect of Pt-drugs alone or with other therapies. Based on this feature of Pt(IV), Wang and co-workers reported a self-assembled nanoprodrug platform (siBec1@PPN) co-delivering cisplatin prodrug and *Beclin1* (autophagy initiation factor) gene-targeted siRNA for synergistic chemotherapy and RNAi (Fig. 7a) [162]. Three chemical modules made up the nanoprodrug platform: DSPE-PEG, cRGD-modified DSPE-PEG (DSPE-PEG-cRGD), and Pt(IV)-peptide-bis(pyrene). As expected, the DSPE-PEG and DSPE-PEG-cRGD could improve the tumor targeting capacity and biocompatibility of the nanoprodrug. After endocytosis by cancer cells, the Pt(IV) could respond to intracellular high-level GSH and be reduced to active Pt(II). This decomposition process could promote the disassociation of the nanoprodrug platform, increasing the siRNA release. In turn, the siRNA-mediated autophagy inhibition could further enhance the Pt(II)-induced apoptosis. As anticipated, the siBec1@PPN could greatly increase autophagy inhibition, intracellular GSH scavenging, and apoptosis in Pt-resistant A549 cells (Fig. 7b-d). Thus, the developed nanoprodrug platform could significantly suppress the growth of cisplatin-resistant tumors in vivo (Fig. 7e).

**Fig. 8:**
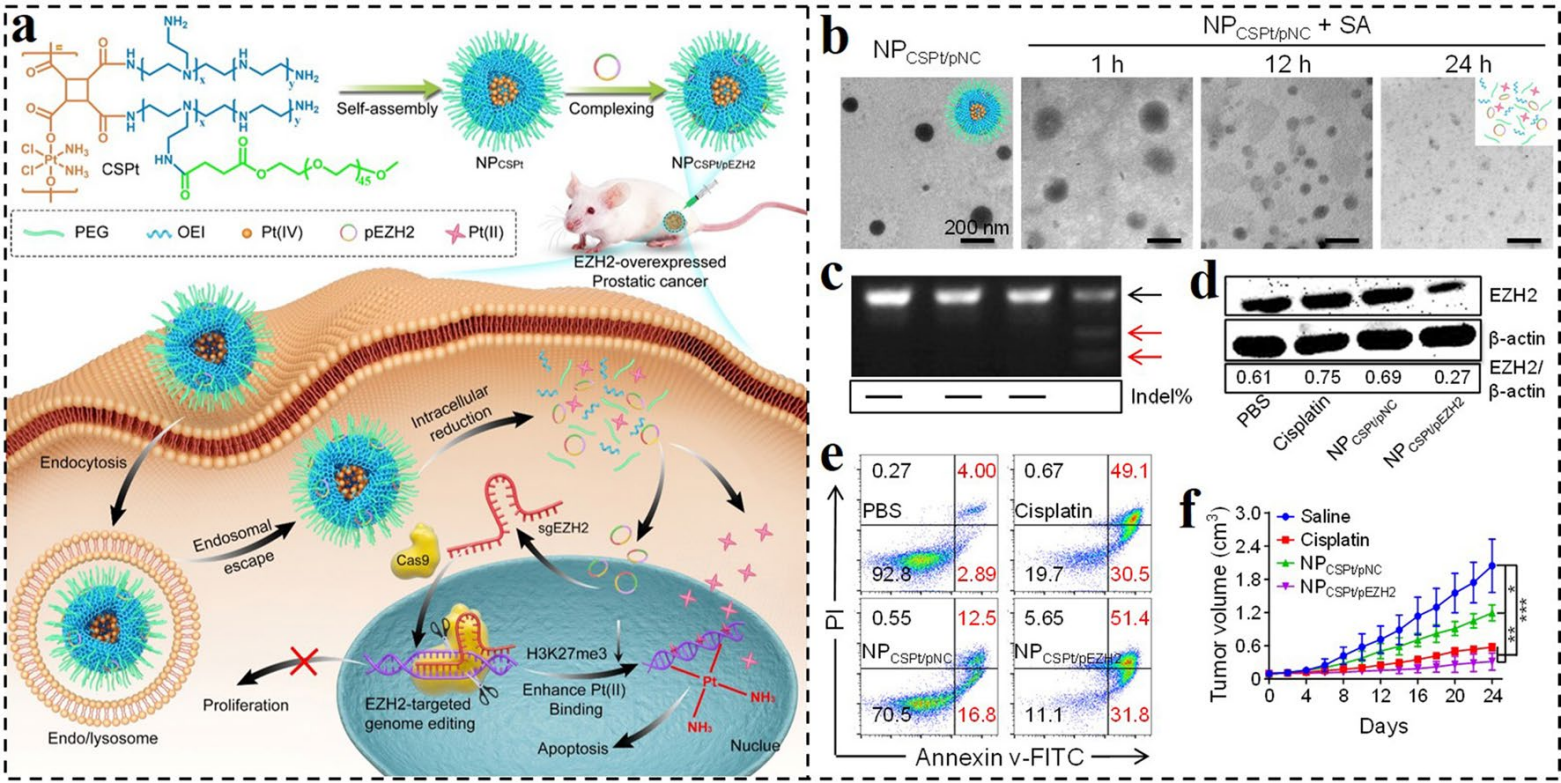
**a** Schematic illustration of the chain-shattering Pt(IV)-backboned polymeric nanoplatform delivering EZH2-targeted CRISPR/Cas9 system (NP_CSPt/pEZH2_) for effective gene editing-chemo synergistic treatment of prostate cancer. **b** Morphology changes of NP_CSPt/pNC_ after incubation with ascorbic acid for different time intervals. **c** Mutation frequency analysis of the targeted sites of EZH2 by T7EI assay. **d** EZH2 protein expression levels in PC-3 cells by Western blot assay after different treatments. **e** Apoptosis analysis of PC-3 cells by flow cytometry after different treatments. **f** Tumor volume changes of mice during the experiment [163]. Copyright 2020, Tsinghua University Press and Springer-Verlag GmbH

By using Pt(IV) not only as a prodrug molecule and the responsive group but also as a polymeric monomer, Zhang et al. developed a Pt(IV)-backboned polymer-based nanocarrier for CRISPR/Cas9 system delivery (NP_CSPt/pEZH2_) and combinational genome editing-chemo of cancer (Fig. 8a) [163]. Tactfully, the Pt(IV) as a monomer was used to synthesize the Pt(IV)-backboned polymer, and then the low toxic OEI_1.8 k_ was introduced to the side chain. The obtained amphiphilic polymer could self-assemble into micellar nanoparticles with the cationic surface, which could bind negatively charged CRISPR/Cas9 plasmid to form the NP_CSPt/pEZH2_. The nanoplatform could be effectively endocytosed by tumor cells and then escaped from the endosomes via the PSE. Due to the reduction-sensitive of Pt(IV), the Pt(IV)-backboned will be specifically cleaved in a chain-shattering pattern under the high level of GSH, promoting the unpack/release of plasmid/Pt(II) to perform their functions (Fig. 8b). This is the first example that the combination of CRISPR/Cas9 system and Pt-drug chemotherapy was achieved by the designed co-delivery nanocarrier. The developed nanoplatform displayed efficient EZH2 gene disruption and corresponding protein downregulation (Fig. 8c, d), leading to substantial suppression of cancer cells in vitro and in vivo (Fig. 8e, f). Thus, the developed nanocarrier simultaneously incorporating CRISPR/Cas9 system and chemotherapeutics could address the safety problem and low gene packaging ability of viral carriers and further achieve gene editing-chemo combination for tumor therapy.

### ROS-Responsive GDNCs

**Fig. 9:**
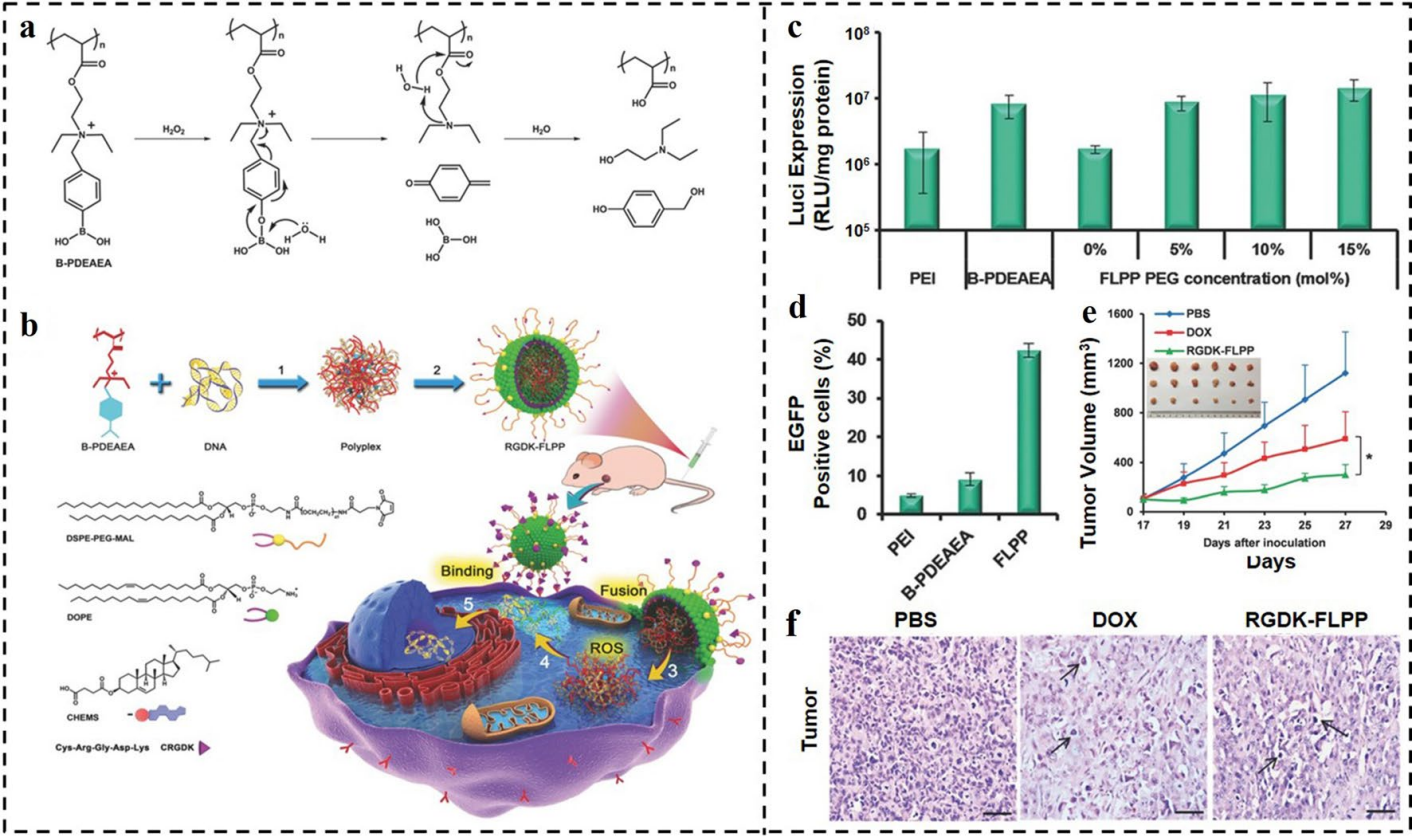
**a**,** b** Schematic representation of ROS-responsive charge-reversal polymer and its CRGDK-receptor targeted fusogenic lipopolyplex (RGDK-FLPPs) for systemic gene delivery. **c** Luciferase gene transfection efficiency of polyplexes in A549 cells after 48 h. **d** Enhanced green fluorescent protein (EGFP) gene transfection efficiency in A549 cells of FLPPs in terms of GFP-positive cell percentage measured by flow cytometry. **e** Antitumor activity of the TRAIL plasmid-loaded RGDK-FLPPs. **f** Representative histological features of tumors [165]. Copyright 2015, WILEY–VCH GmbH

In comparison with pH- and GSH-responsive DDSs, the ROS-responsive nanocarriers have higher selectivity in cancer cells. In response to the high content of ROS in tumor cells, ROS-sensitive linkage would be quickly broken and the rupture of the carrier will promote the release of the internal cargo [164]. For example, Shen and co-workers developed a polymer of poly[(2-acryloyl)ethyl(p-boronic acid benzyl)diethylammonium bromide] (B-PDEAEA) with ROS-labile charge-reversal character for gene complexing and forming the polyplexes (Fig. 9a, b) [165]. To avoid endocytosis, trafficking into lysosomes, and encapsulated DNA degradation, the polyplexes were further coated with lipid envelope to form virus-mimicking fusogenic lipopolyplexes (FLPPs). The tumor-homing specific peptide CRGDK and PEGylation allowed the virus-mimicking FLPPs to stay stable in the bloodstream and efficiently accumulate in the tumor. The FLPPs could act like a paramyxovirus to coalesce with cytomembrane, and then eject the complex into the cytoplasm, avoiding the lysosome trapping. In the cytosol, the boronic acid unit of B-PDEAEA would be oxidated by ROS, changing the quaternary ammonium to tertiary amine and further self-catalyzing fast hydrolysis to produce negative poly(acrylic acid). Thus, the ROS-responsive polyplexes with charge-reversal features could be efficiently dissociated to unpack the gene into the cytosol, promoting gene transfection. The studies displayed that FLPPs had better gene transfection efficiency than PEI_25k_ (gold standard). As shown in Fig. 9c, the luciferase expression level in the FLPPs group was nearly ten-fold higher than that of the PEI_25k_ group. Remarkably, compared to PEI_25k_ and non-coated B-PDEAEA, the FLPPs transfected cells far more effectively (Fig. 9d). The FLPPs loaded with the TRAIL gene significantly inhibited the tumor growth, evidently more effectively than traditional chemotherapeutic drug doxorubicin (Fig. 9e, f). Collectively, the developed fusogenic ROS-triggered charge-reversal vector showed great promise in gene therapy.

**Fig. 10:**
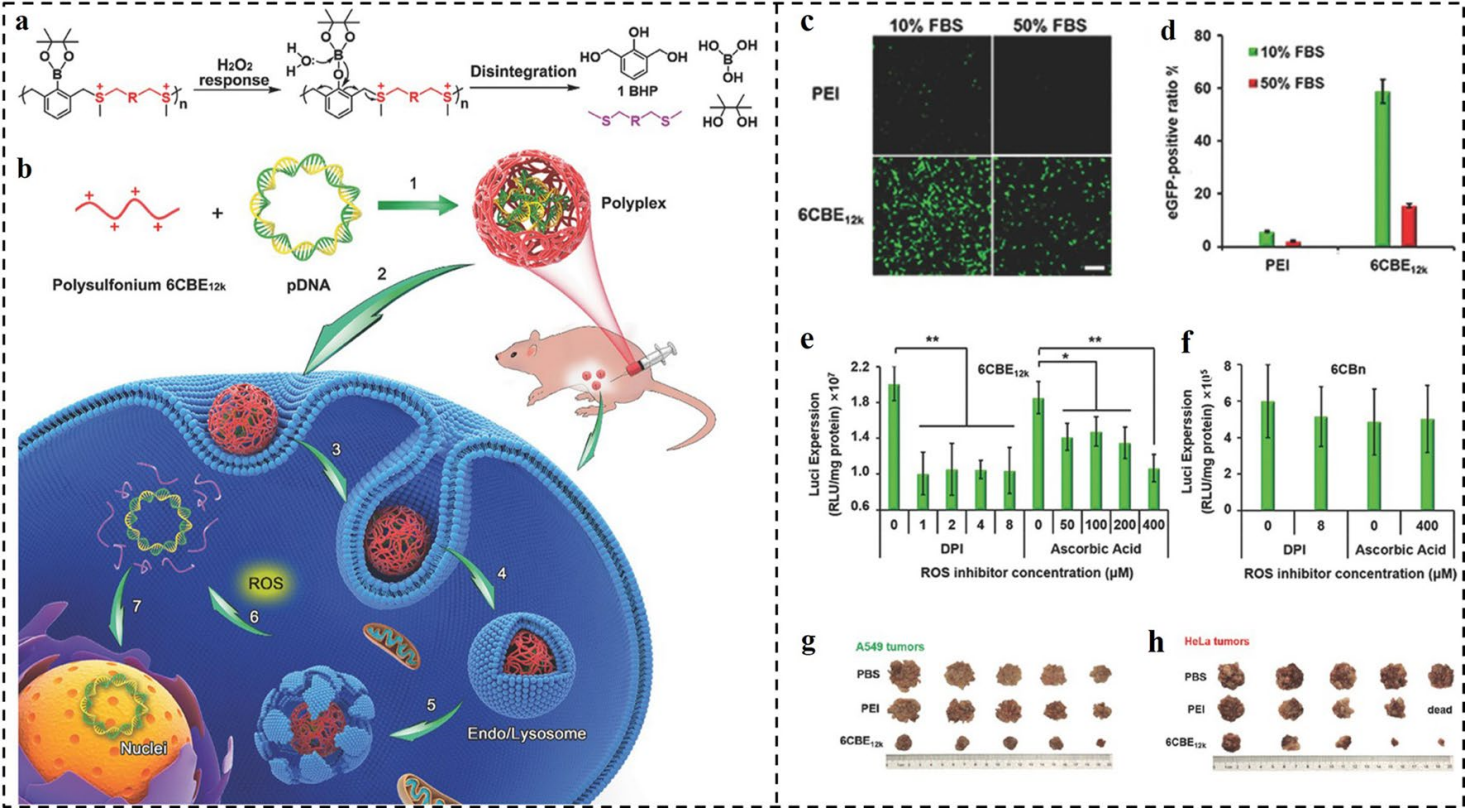
**a**,** b** ROS-responsive disintegrable polysulfonium and its polyplex for intraperitoneal gene delivery. **c**,** d** Comparison of EGFP expression mediated by PEI_25k_ (*N/P* = 7) or 6CBE_12k_ (*S/P* = 60) polyplexes. **e**,** f** Effects of DPI on luciferase expression mediated by **e** ROS-responsive 6CBE_12k_ polyplexes (*S/P* = 60) and **f** ROS-insensitive 6CBn polyplexes (*S/P* = 8). **g**,** h** Images of A549 and Hela tumors in each group [170]. Copyright 2017, WILEY–VCH GmbH

Although amine-contained cationic macromolecules have been widely investigated as non-viral vectors, gene release from polyplexes in cells remains a key barrier to a high rate of transfection and expression [166]. Besides, the inherent positively charged polymer chain caused by the amine, as well as those of their degraded fragments, prevents transgene expression [167, 168]. In addition to quickly releasing the gene, a cationic polymer can break down into uncharged fragments inside cells without hindering gene transcription, allowing for efficient gene expression [169]. According to this principle, in another work, Shen and co-workers developed ROS-responsive disintegrable polysulfoniums for efficient gene delivery (Fig. 10a, b) [170]. The cationic polysulfonium could compact genes into nano-polyplexes that could be efficiently internalized and escape from endosomes. Thereafter, the polysulfonium degraded into neutral thioether pieces initiated by high-concentration ROS, promoting the unpacking of loaded genes to enhance transcription. The polyplexes had excellent serum resistance. In 10% fetal bovine serum (FBS), the polysulfonium 6CBE_12k_ complexes could infect 58.8% of cells, and even 15.5% of infected cells could be observed in 50% of FBS medium. By stark contrast, in the 10% FBS medium, PEI_25k_/pEGFP transfected only 5.7% of cells, and this quantitative value is hardly observed in the 50% FBS condition (Fig. 10c, d). After being pre-treated with ROS scavengers diphenylene-iodonium (DPI) and ascorbic acid, the 6CBE_12k_ polyplexes-mediated luciferase expression significantly decreased to about 50%, while the transfection efficiency of ROS-insensitive 6CBn polyplexes was negligible changed (Fig. 10e, f). Obviously, the transfection efficiency is highly dependent on ROS. The in vivo results indicated that the 6CBE_12k_/pTRAIL polyplexes can remarkably suppress the growth of A549 and Hela tumors (Fig. 10g, h). Taken together, the disintegrable polysulfonium displayed great potential in gene delivery for tumor therapy.

**Fig. 11:**
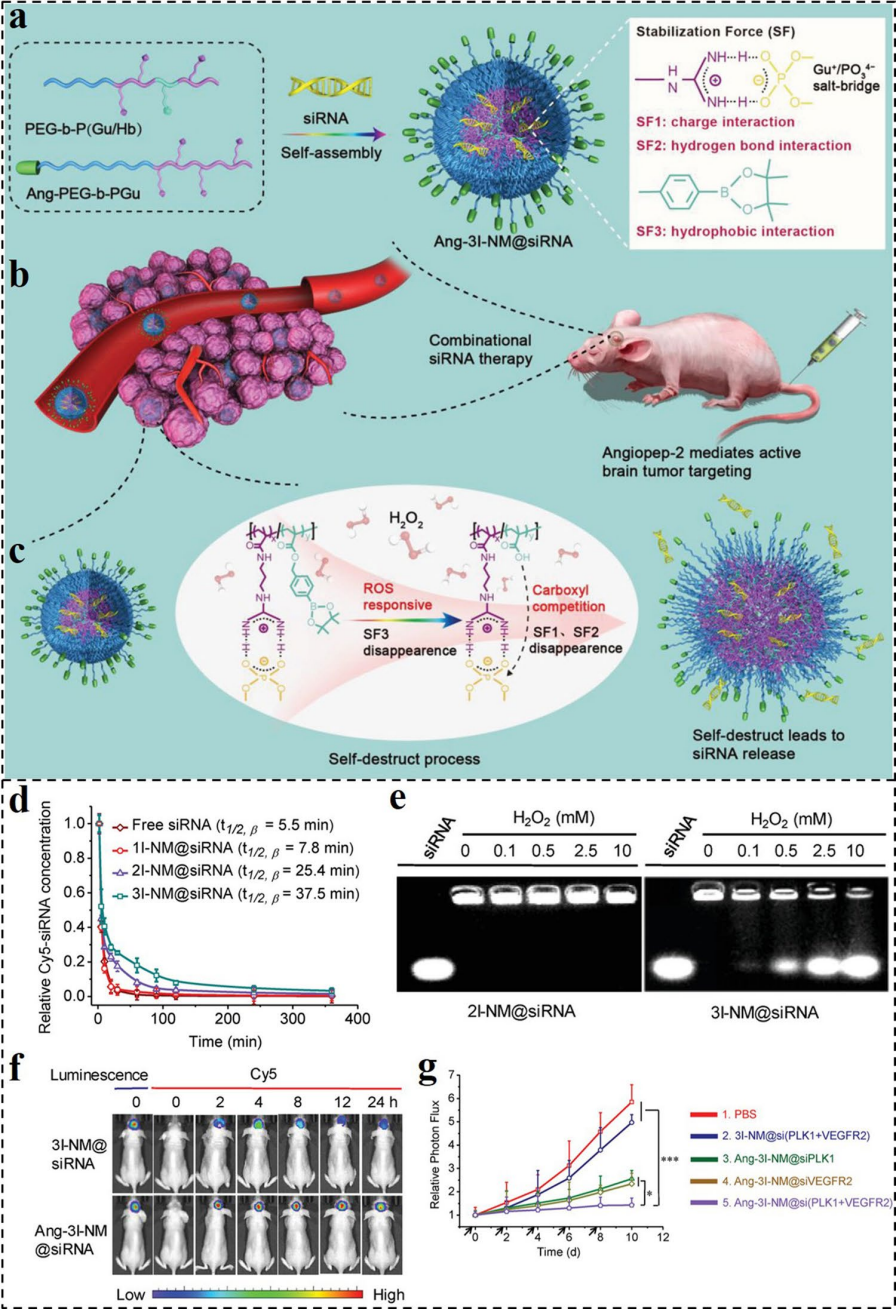
**a** Schematic illustration of the formation of Ang-3I-NM@siRNA stabilized by the three “triple-interaction” forces, namely, electrostatic, hydrogen bond, and hydrophobic interactions. **b** Active tumor uptake and combinational RNAi therapy. Angiopep-2 ligand enables nanomedicines to actively cross the BBB to target GBM. **c** Mechanisms of ROS triggered siRNA release. **d** In vivo pharmacokinetics of 1I-NM@siRNA, 2I-NM@siRNA and 3I-NM@siRNA, and free siRNA in mice. **e** siRNA release from 3I-NM@siRNA following H_2_O_2_ treatment. **f** Fluorescence images of orthotopic U87MG-Luc human glioblastoma tumor-bearing nude mice at different time points following injection of Ang-3I-NM@siRNA. **g** Quantified the luminescence levels of mice using the Lumina IVIS III system after the mice were treated with different drugs [171]. Copyright 2019, WILEY–VCH GmbH

Although RNAi technology holds great potential in treating various tumors, their applications are remaining daunting challenges of lacking suitable delivery vectors with excellent circulation stability and site-specific delivery ability. Besides, strengthening the stability of siRNA nanomedicines under biological environments often causes the unintended consequence of impairing intracellular siRNA release, which in turn impairs RNAi performance. To overcome this dilemma, Shi and co-workers developed a ROS-responsive nanocarrier (3I-NM@siRNA) for effective siRNA delivery. The 3I-NM@siRNA was stabilized by hydrogen bond, electrostatic, and hydrophobic triple interactions (Fig. 11a–c) [171]. Compared with the sole electrostatic interaction in common siRNA nanomedicines, the hydrophobic and hydrogen bond interactions further multiplied the stability of the developed nanomedicine, making it stable in vivo during blood circulation. In addition, Ang ligand decorated Ang-3I-NM@siRNA enabled the target to GBM due to the superb BBB penetration ability. Importantly, the hydrophobic phenylboronic ester in the nanomedicine would convert to the hydrophilic counterpart with carboxyl groups in cancer cells with high-level ROS, enabling “self-destruct” siRNA release. In vitro results revealed that the 3I-NM@siRNA with triple interactions had the best stability compared with the 1I-NM@siRNA and 2I-NM@siRNA nanomedicines with sole and dual interactions, respectively (Fig. 11d). Under the ROS condition, the 3I-NM@siRNA disintegrated and subsequently released siRNA, while the ROS-insensitive 2I-NM@siRNA had not been influenced (Fig. 11e). After the Ang decorating, the Ang-3I-NM@siRNA displayed remarkably higher BBB penetration ability and enhanced accumulation in orthotopic GBM brain tumors (Fig. 11f). By integrating siRNAs targeting polo-like kinase 1 (PLK1) and vascular endothelial growth factor receptor-2 (VEGF2), the Ang-3I-NM@siRNA effectively suppressed the GBM tumor growth (Fig. 11g). Thus, the reported siRNA nanomedicine with triple-interaction stabilization combined with built-in “self-destruct” delivery capabilities provided a reliable and effective strategy for gene delivery in cancer therapy.

### Light-Responsive GDNCs

**Fig. 12:**
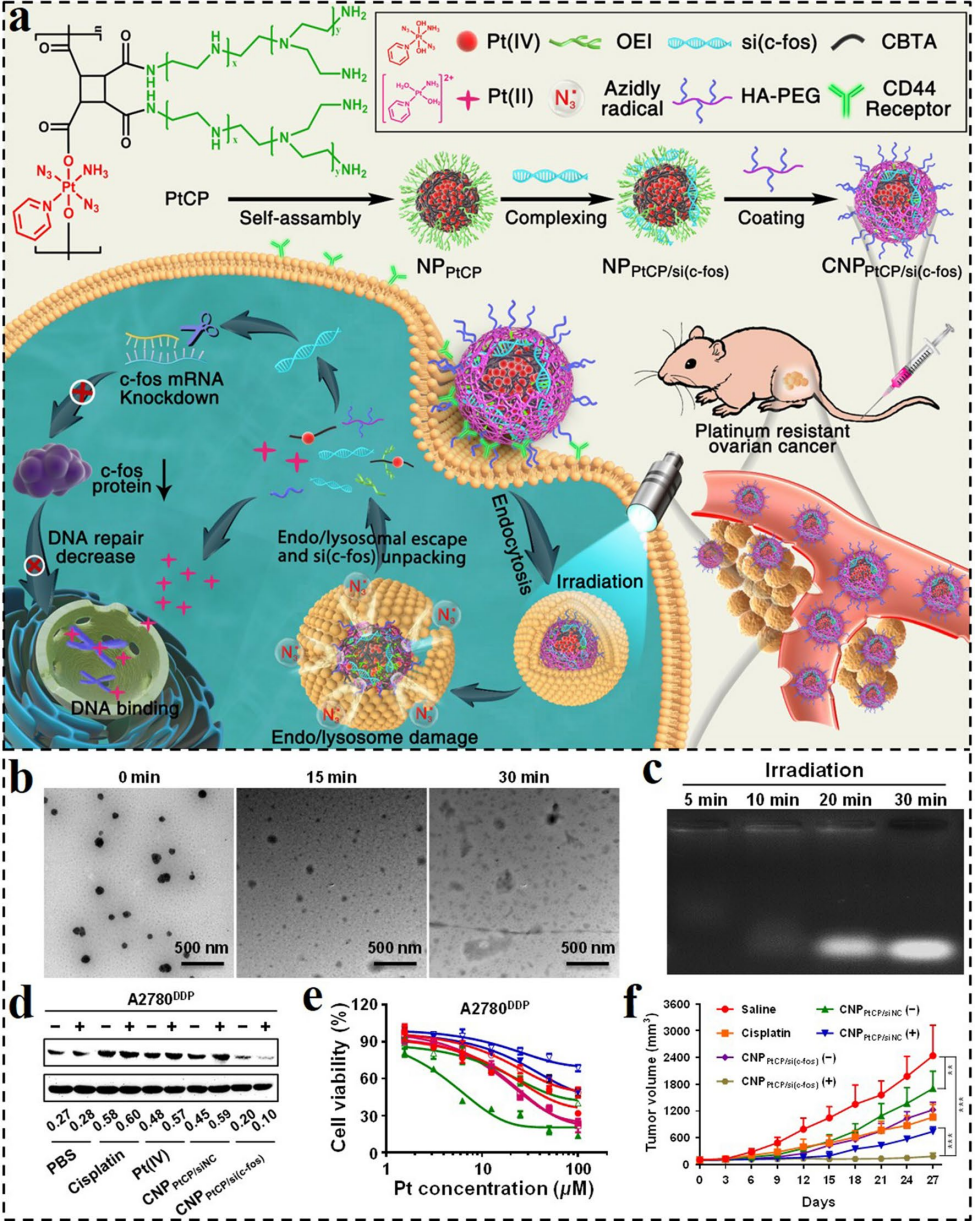
**a** Schematic illustration of the photoactivatable Pt(IV) prodrug-backboned polymeric nanoparticle system (CNP_PtCP/si(c‑fos)_) for light-controlled efficient gene delivery and synergistic chemotherapy and RNAi on PROC. **b** Morphology changes of CNP_PtCP/si(c‑fos)_ after irradiation for different time intervals. **c** Photo-responsive dissociation of CNP_PtCP/si(c‑fos)_ after irradiation for different time intervals. **d** c-fos protein expression levels of A2780^DDP^ cells after different treatments. **e** Cell viability curves of A2780^DDP^ after incubation with different drugs for 72 h with or without irradiation. **f** Tumor volume changes of mice during the experiment [174]. Copyright 2020, American Chemical Society

Light as a therapeutic and diagnostic tool has been extensively used in treating various diseases [172, 173]. Recently, light-regulative nanomedicines provide alternative options for biomolecule delivery with high spatiotemporal precision. By introducing photo-responsive groups or molecules into nanomedicine, photoactivatable GDNCs could be obtained for efficient gene delivery. For instance, Huang et al. designed a photo-responsive nanosystem (CNP_PtCP/si(c‑fos)_) for controllable siRNA delivery, and synergistic RNAi and chemotherapy against platinum-resistant ovarian cancer (PROC) (Fig. 12a) [174]. Innovatively, the azide radicals (N_3_^•^) generated from Pt(IV) prodrug instead of ROS were applied for assisting the lysosomal escape of the nanosystem. Subsequently, the photo-reduction of Pt(IV) would induce the main-chain cleavage, resulting in the disintegration of CNP_PtCP/si(c‑fos)_ and the release of active Pt(II) and si(c-fos) concurrently. As shown in Fig. 12b, c, the nanosystem evidently fell apart into pieces under blue light irradiation, and the complexed siRNA was gradually released with the extension of irradiation time. The photo-responsive CNP_PtCP/si(c‑fos)_ remarkably downregulated the expression of the target gene of c-fos, displayed potent cytotoxicity against Pt-drug resistant tumor cells in vitro, and efficiently controlled the growth of PROC in vivo (Fig. 12d-f).

Although with good treatment effects, the developed blue light-activated co-delivery nanosystems are limited by the penetration depth, which is not in favor of precise spatiotemporal manipulations of gene delivery. Herein, Huang and colleagues developed another upconversion nanoparticle (UCNP)-based polyprodrug/siRNA co-delivery nanoplatforms. This nanoplatform has near-infrared light (NIR)-activatable peculiarity, and upconversion luminescence, magnetic resonance, and computed tomography tri-modal imaging ability simultaneously [124]. In this work, the Pt(IV)-backbone polymer (PPt) was linked to the PEI-coated UCNPs, and then complexed PLK1-targeted siRNA via electrostatic interaction to obtain multi-functional NIR-responsive codelivery system (PUCNP@Pt@siPLK1). As a nanotransducer, the UCNPs can transform 980 nm NIR light into UV light for the breakage of PPt, promoting the release of Pt(II) and siPLK1 concurrently. In addition, the PUCNP@Pt@siPLK1 with tri-modality imaging ability would guide the NIR light-activated chemotherapy and RNAi, possessing great promise in treating various cancers.

**Fig. 13:**
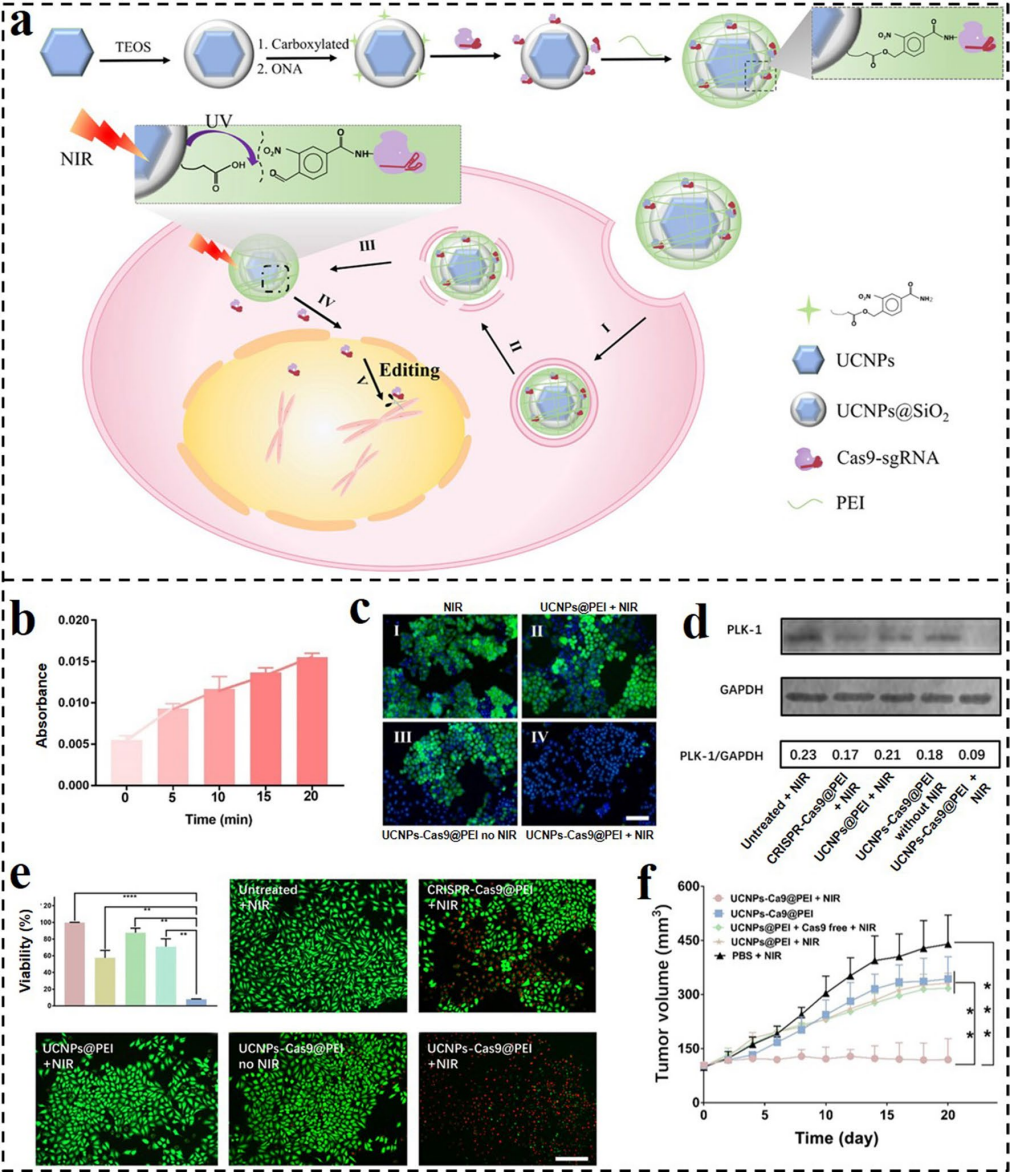
**a** Preparation of UCNPs-Cas9@PEI and the NIR-triggered delivery of Cas9-sgRNA to the nucleus of the cell for gene editing. **b** UV–Vis absorption of the supernatant of UCNPs-Cas9 with different irradiation times by a 980 nm laser. **c** Fluorescence microscopy images of EGFP in KB cells treated with different formulations. **d** Western blot assay of PLK1 protein after A549 cells treated with different formulations. **e** CCK-8 and live/dead staining analyses of A549 cells treated with different formulations. **f** Tumor sizes after different treatments [176]. Copyright 2019, American Association for the Advancement of Science

CRISPR–Cas9 system is an innovative technique in the treatment of various diseases [175]. Nowadays, nanomaterial-based non-viral vectors have been extensively designed for CRISPR–Cas9 delivery. However, the controllable CRISPR-Cas9 release allowing accurate remote spatiotemporal control over genome editing is still hard to accomplish. Therefore, Song and co-workers designed a UCNP-based nanoplatform for NIR light-activated CRISPR–Cas9 system and cancer therapy (Fig. 13a) [176]. By using photocleavable 4-(hydroxymethyl)-3-nitrobenzoic acid (ONA), CRISPR-Cas9 was covalently attached to UCNPs and subsequently coated with PEI to obtain the nanoplatform (UCNPs-Cas9@PEI). Upon 980-nm irradiation, UV light can be generated via the upconversion ability of UCNPs, which induced the cleavage of ONA and then promoted the release of the CRISPR-Cas9 system (Fig. 13b). EGFP was chosen as a model protein that can reveal the gene editing efficacy of UCNPs-Cas9@PEI. The green fluorescence intensity in UCNPs-Cas9@PEI treated cells with 980-nm irradiation was remarkably decreased in comparison with other groups (Fig. 13c). Similarly, the UCNPs-Cas9@PEI-mediated PLK1 expression was remarkably less than other groups (Fig. 13d). The developed NIR-activated nanoplatform substantially inhibited the proliferation of cancer cells and tumor growth (Fig. 13e, f).

### Other-Responsive GDNCs

**Fig. 14:**
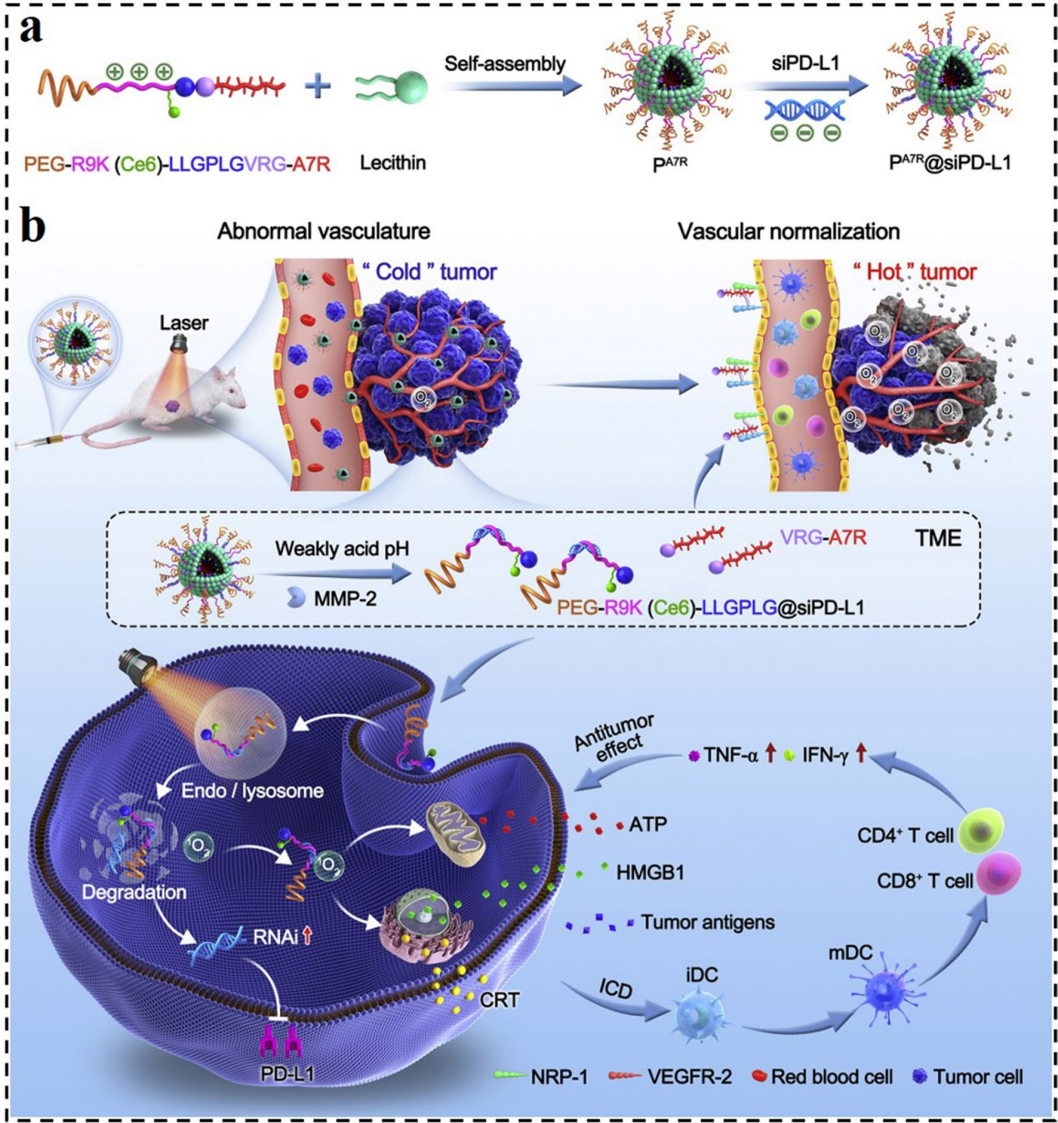
Illustration of MMP-2-responsive, peptide-assembled micelleplexes for enhanced photoimmunotherapy. **a** Composition and self-assembly process of P^A7R^@siPD-L1. **b** Antitumor mechanism of PA7R@siPD-L1-mediated photoimmunotherapy [179]. Copyright 2022, Elsevier

Enzymes are an important class of biocatalysts involved in almost all biological and metabolic processes [177]. Enzymes enable chemical processes in living organisms to proceed precisely and effectively even in relatively mild circumstances. However, enzyme dysfunction would lead to the overexpression of various enzymes in tumor tissues. By utilizing this feature, enzyme-responsive GDNCs have recently become a research focus in cancer therapy, which offered a potential technique to improve therapeutic efficiency [178]. In one case, Tao et al. conceived a strategy of turning “cold” tumors into “hot” ones by the developed MMP-2-responsive siRNA micelleplexes (P^A7R^@siPD-L1, Fig. 14) [179]. The micelleplexes were assembled through hydrophobic interaction between chimeric peptide and lecithin and further bounded with siRNA target to PD-L1. In the chimeric peptide, the A7R (an anti-angiogenic peptide) was linked to the chlorin e6 (Ce6)-conjugated PEG-R9 peptide through the MMP-2 substrate spacer (GPLGVRG). Therefore, the micelleplex structure of P^A7R^@siPD-L1 would be gradually disintegrated when exposed to high-level MMP-2 in the TME. Subsequently, siPD-L1 along with PEGR9K(Ce6)-LLGPLG could respond to release from the micelleplexes and then enter cancer cells with the help of cell-penetrating component R9. Under light irradiation, the Ce6 could produce ROS, which could not only be used for PDT and trigging immunogenic cell death but also induced the disruption of lysosome to facilitate siPD-L1 diffusion to the cytoplasm. During the dissociation of P^A7R^@siPD-L1, the A7R could respond to release and target VEGF receptor 2 and neuropilin-1 on the surface of tumor endothelial cells. It would result in the normalization of blood vessels, the alleviation of hypoxia, and the reduction of immunosuppression TME. As a result, the developed MMP-2 responsive micelleplexes displayed strong and durable antitumor immunity, significantly inhibiting tumors' recurrence and metastasis. These features indicated that the stimulus-responsive micelleplex is a promising universal delivery nanocarrier for loading various RNA molecules in treating a lot of malignancies.

**Fig. 15:**
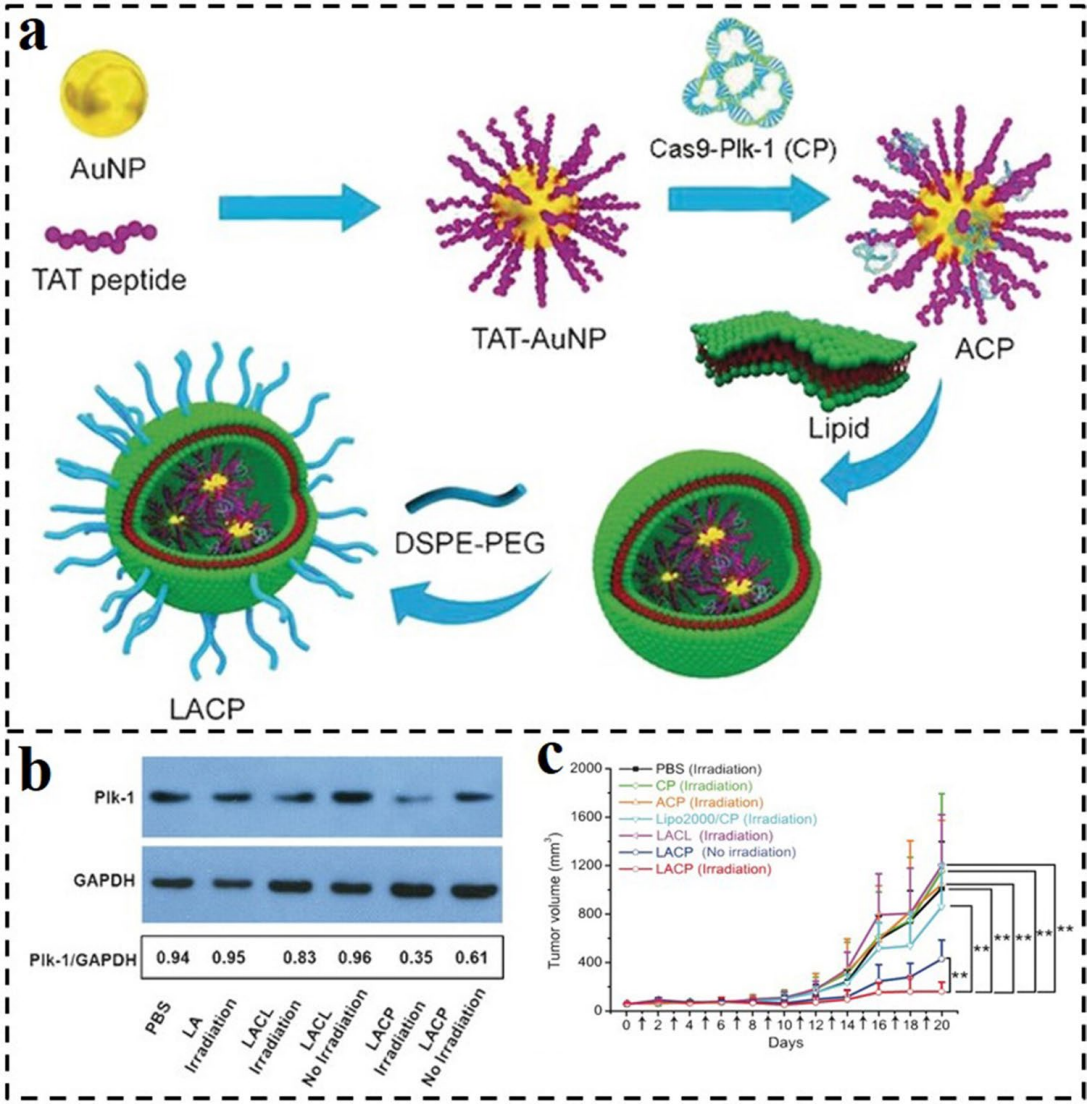
**a** Synthesis process for LACPs. **b** Western blot analysis of the Plk-1 protein expression in A375 cells treated by different CP formulations with or without laser irradiation. **c** Sizes of tumors treated with different CP formulations [180]. Copyright 2019, Wiley–VCH GmbH

Photothermal response manipulation of biomolecule delivery is a promising method in biomedicine applications. To exploit an effective CRISPR/Cas9 delivery carrier, Jiang et al. constructed a thermal-responsive lipid-encapsulated gold nanoplatform (LACP). Here, Cas9-sgPlk-1 plasmids (CP) were complexed to TAT (GRKKRRQRRRPQ) peptide-modified AuNPs via electrostatic adsorption and then coated with lipids to form the LACP (Fig. 15a) [180]. The lipid out-layer endows the LACP with high stability and promotes its cellular internalization. Specifically, the AuNPs inner core could generate local heat under NIR laser irradiation, triggering the release of a loaded CRISPR-Cas9 system. As investigated by Western blot assay, Cas9-sgPlk-1 laden LACP induced 40% downregulation of Plk-1 protein, which was significantly increased to 65% via photothermal triggering (Fig. 15b). In vivo tumor inhibition results demonstrated that the LACP treatment induced an 85% tumor decrease when laser irradiation was applied (Fig. 15c). Thus, LACP is a promising system for treating tumors due to the synergistic effects of photothermal-mediated cytoplasm liberation of CP and consequent CP-induced apoptosis.

**Fig. 16:**
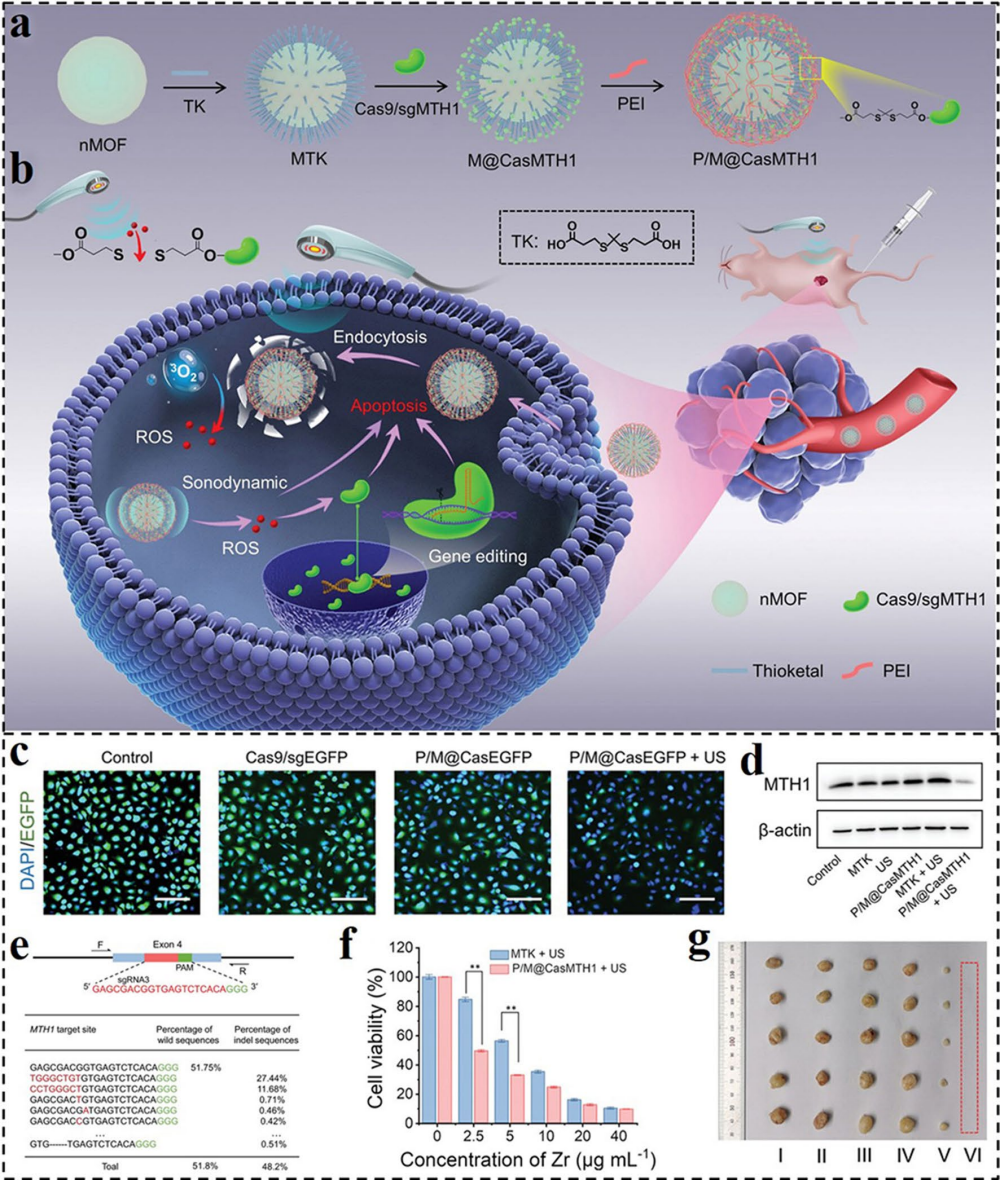
Schematic illustration of **a** fabrication of the nanosonosensitizer P/M@CasMTH1 and **b** US-triggered genome-editing-augmented SDT for tumor treatment. **c** Confocal laser scanning microscopy (CLSM) images of A549-EGFP cells after different treatments. **d** Western blot analysis of the MTH1 expression in A549 cells after different treatments. **e** NGS analysis of indel percentage in P/M@CasMTH1 or P/M@CasMTH1 + US treatment groups. **f** Viability of A549 cells after SDT or combined therapy treatment. **g** Digital photographs of dissected tumors in different treatment groups [181]. Copyright 2021, Wiley–VCH GmbH

As a mechanical wave, the US can reach deeply into tumor sites, focus energy, and initiate sonosensitizers to produce harmful ROS, especially ^1^O_2_. Currently, US-mediated SDT has been thoroughly studied. Owing to the spatiotemporal controllability, US regulation provides a noninvasive and precisely controlled means for bioactive drug/protein/gene delivery in tumor tissues. For example, Xu and colleagues reported a sono-controllable and ROS-responsive nanosonosensitizer (P/M@CasMTH1) for on-demand delivery of CRISPR-Cas9 system and synergistic enhancing therapeutic with SDT (Fig. 16a, b) [181]. CRISPR-Cas9 ribonucleoprotein (RNP) was covalently bonded to ROS-responsive thioether bonds on the surface of nanoscale metal–organic frameworks (nMOFs), and then coated with PEI to obtain the P/M@CasMTH1. Under US irradiation, sufficient ROS would generate in situ after internalization by tumor cells to trigger the release of the loaded RNP, inducing MTH1 gene disruption via the CRISPR-Cas9 system. The self-defense mechanism of tumor cells against ROS would be damaged by the knockout of MTH1. As a proof of concept, the P/M@CasEGFP and P/M@CasMTH1 can conspicuously suppress the expression of EGFP and MTH1 proteins under US irradiation, respectively, as seen in Fig. 16c, d. The effectiveness of MTH1 gene disruption was also assessed by next-generation sequencing (NGS). The gene disruption efficiency of the P/M@CasMTH1 + US treated group was 48.2%, whereas the efficiency in the P/M@CasMTH1-treated group was much lower (10.8%), highlighting the importance of the US as an on–off for the regulated gene therapy (Fig. 16e). Based on these efficacies, the P/M@CasMTH1 could significantly induce apparent apoptosis of cells and prominent tumor inhibition through gene-editing-enhanced SDT (Fig. 16f, g).

**Fig. 17:**
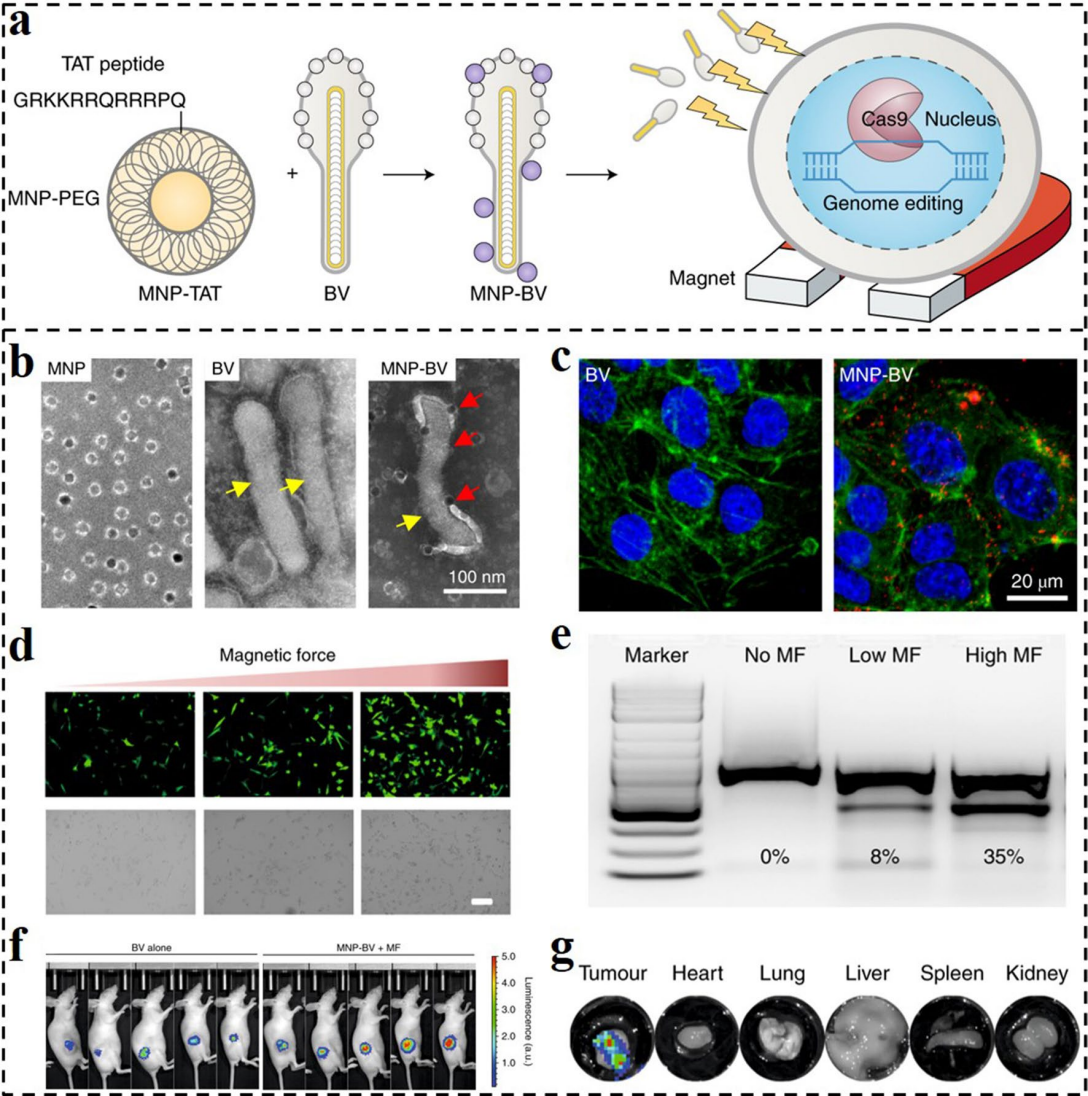
**a** MNPs are coated with PEG, and conjugated with TAT to facilitate the interaction with BVs. An external MF improves the entry of MNP–BVs carrying the CRISPR–Cas9 system into cells [184]. Copyright 2019, Springer Nature. **b** TEM images of MNPs, BV and MNP-BV. **c** MNPs under external MF enhanced endocytosis of BV. **d** Transgene expression was examined by EGFP fluorescence at 24 h after transduction. **e** CRISPR-mediated VEGFR2 disruption correlated with the MF strength. **f** Bioluminescence analysis of transgene expression in the tumor at 24 h post-transduction. **g** Bioluminescence analysis of transgene expression in the dissected tumors and vital organs. **b-g** Reproduced with permission from Ref. [137]. Copyright 2019, Springer Nature

The MF is another commonly used exogenous stimulus for spatiotemporal control and noninvasive manipulation of drug/gene/protein delivery. External MF gradients enable delivery systems to be steered toward specific locations, allowing remote-controlled targeted drug delivery owing to their outstanding tissue penetrating properties [182]. MF-guiding of CRISPR-Cas9 in the field of genome editing can reduce the risk of undesirable off-target consequences, making precise genome editing achievable [183]. To this end, Bao and co-workers developed a hybrid MF-responsive gene delivery system using a baculoviral vector (BV) complexed with magnetic iron oxide nanoparticles (MNP-BV) that enables spatial control of in vivo genome editing [137]. The MNPs were first coated with co-polymers of phospholipid and PEG, and then the nanoparticles were coupled with a TAT peptide to facilitate their interaction with BVs (Fig. 17a, b) [184]. The MF-responsive MNP-BV could enhance the cellular uptake of MNPs and BV in vitro under an MF (Fig. 17c). The large size of BVs allowed for a high loading capacity of Cas9/sgRNA DNA and enabled powerful yet transient gene expression (Fig. 17d). The external MF could be regarded as the "on" button to make it easier for the MNP-BV complex to enter cells locally for tissue-specific genome editing, and the BVs inactivation could be regarded as the "off" switch to limit the systematic actions of genome editing. They administered a BV intratumorally and discovered that BV-LUC could moderately promote transgenic expression in subcutaneous tumors. In contrast, MNP-BV-LUC injection into the tumor followed by 1 h of exposure to an MF significantly boosted transgene expression (Fig. 17e). Dissected tumors and major organs were imaged ex vivo, and the results revealed that the transgenic expression was only present in the injected tumor tissue (Fig. 17f, g). Thus, the MNP-BV-based delivery system has the potential to facilitate controllable genome editing in vivo for cancer therapy.

**Fig. 18:**
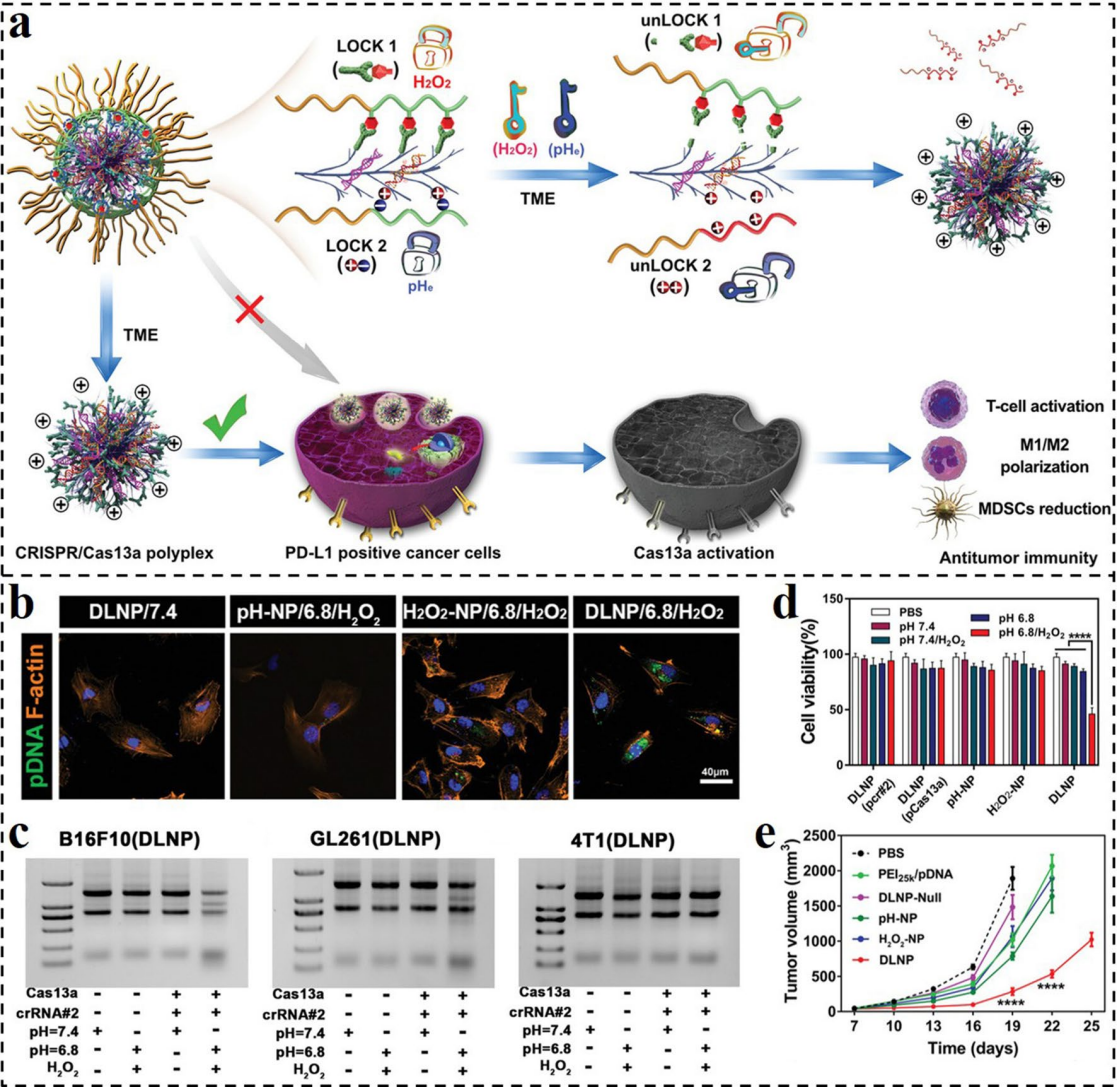
**a** Schematic representation of DLNP for effective cancer immunotherapy. Similar to dual-lock safes that can only be opened when both locks are unlocked, DLNP can only release the CRISPR/Cas13a system in a microenvironment where both a low pH_e_ and high H_2_O_2_ concentration are present. **b** CLSM analysis of intracellular delivery of different formulations to U87MG cells after 2 h of incubation under different conditions. **c** RNA denaturing gel of B16F10, GL26, and 4T1 cells after being treated with DLNP at different conditions for 60 h. **d** Cell viability of B16F10 cells after being treated with different formulations under different conditions for 60 h. **e** Average tumor growth kinetics in different groups [186]. Copyright 2019, Wiley–VCH GmbH

To achieve more precise control of gene delivery, dual- or multi-responsive nanocarrier provides an effective strategy. For example, CRISPR-Cas13a displays significant potential for cancer therapy because of its capability of targeting tumor cells [185]. However, due to a lack of targeting specificity, the systemic administration of the Cas13a/PD-L1-based agent might lead to unexpected cell death in normal tissues. Herein, Liu and co-workers described a dual-locking nanoparticle (DLNP) responding to low pH and high H_2_O_2_ concentration for specific activation CRISPR/Cas13a in tumor tissues for immunotherapy (Fig. 18a) [186]. Although many pH- or ROS-responsive nanocarriers were developed for nucleic acid drug delivery, it is still hard to satisfy the precisely controlled activation of gene therapy merely relying on a single stimulus. By contrast, this work provides a universal platform for the dual-responsive delivery of genes in tumor tissues with high safety and efficiency. The negatively charged and PEGylated polymer out-layer endowed the DLNP circulation stability and facilitated CRISPR/Cas13a internalization and activation in tumors. As shown in Fig. 18b, more fluorescence could be observed in DLNP-treated cells at dual stimulation of pH 6.8/H_2_O_2_, compared with those in DLNP/pH 7.4 or H_2_O_2_-NP and pH-NP groups with a single stimulus. Only at the condition of pH 6.8/H_2_O_2_, obvious gene editing was observed by the Cas13a/PD-L1 system-loaded DLNP (Fig. 18c). The cell viability assays against B16F10 cells also revealed that only the DLNP treatment at pH 6.8/H_2_O_2_ could induce evident cell viability loss (Fig. 18d). In vivo antitumor results demonstrated that the Cas13a/PD-L1-laden DLNP displayed the best tumor inhibition ability with high safety (Fig. 18e). Taken together, the reported pH and H_2_O_2_ dual-responsive nanocarrier provide a safe and efficient strategy for CRISPR/Cas13a delivery with the ability of precisely controlled activation, an enlightening novel delivery platform for a variety of genes.

## Summary and Perspectives

Gene therapy has attracted extensive interest in the oncotherapy field due to its strong selectivity and effectiveness. In order to protect nucleic acid drugs from premature degradation, prolong the circulation time in blood, achieve the target tumor delivery, as well as improve therapeutic efficiency, numerous stimuli-responsive GDNCs have been developed. Based on a variety of internal and external microenvironments in tumor tissues including acidity, high concentration of GSH and ROS, overexpressed enzyme, etc., corresponding TME-responsive GDNCs have been exploited for tumor-specific response delivery of genes. Meanwhile, by utilizing external triggers such as light, thermal, US, and MF, spatiotemporally controllable GDNCs could be designed for on-demand and site-specific gene release. In this review, the endogenous and exogenous stimuli-responsive GDNCs for gene therapy have been summarized to provide novel perspectives on handling gene delivery and improving therapeutic efficiency.

Despite the significant advances, there are still several pivotal obstacles that require to be overcome to achieve the potential of GDNCs in the clinic application. Firstly, the gene transfection efficiencies of the majority of these newly developed non-viral GDNCs only managed to reach the level that is just higher than the PEI_25K_ polyplex, which is very discouraging [187, 188]. Some were marginally better, but they were still massively inferior to viral vectors, resulting in modest therapeutic effects in vivo [189]. Although nanocarriers can improve tumor accumulation and penetration compared with small molecule drugs, the utilization rate (< 5%) of drugs in nanocarriers is still far from enough [51, 190]. The most crucial point for the systemic delivery of genes by utilizing non-viral nanocarriers is to figure out how to enable more carriers to pass through the vascular endothelium, extravasate out, and further fast disperse away [191]. Furthermore, to enhance the possibility of the clinical application of GDNCs, preclinical animal models that closely resemble the physiological condition of humans still require to be upgraded and standardized [93]. The stability and repeatability of GDNCs are major hurdles to their clinical therapeutic application. There is still a long way to scale up industrial production and balance the complexity of the nanomaterial and the precision toward various stimulations. Taken together, great efforts still need to be devoted to devising more efficient stimuli-responsive GDNCs.

In summary, this review presented the potential and advancement of stimuli-responsive GDNCs to overcome challenges associated with cancer gene therapy. The progresses that have been made by these non-viral nano-vectors provide new chances and directions for the efficient delivery of genes. To achieve more effective controllability and manipulation of gene delivery for better therapeutic potential, the future design and development of stimuli-responsive GDNCs need to require the integration of dual- or multi-responsiveness. In addition, the therapeutic outcome of single gene therapy is often unsatisfactory due to the aberrant expression and mutation of many genes that are involved in the development and progression of cancer. Thus, novel stimuli-responsive GDNCs that could combine gene therapy and other treatment modalities are worth further delving into. The overall objective of this review is to discuss the biomedical potential of stimuli-responsive GDNCs for cancer therapy, along with guidance for developing novel gene delivery nanosystems that are clinically applicable. There is no doubt that gene therapy based on stimuli-responsive GDNCs will bring significant advancements in the treatment of cancers in the near future.
